# Allele-specific expression analysis for complex genetic phenotypes applied to a unique dilated cardiomyopathy cohort

**DOI:** 10.1038/s41598-023-27591-7

**Published:** 2023-01-11

**Authors:** Daan van Beek, Job Verdonschot, Kasper Derks, Han Brunner, Theo M. de Kok, Ilja C. W. Arts, Stephane Heymans, Martina Kutmon, Michiel Adriaens

**Affiliations:** 1 Maastricht Centre for Systems Biology (MaCSBio), Maastricht University, Maastricht, 6229 EN The Netherlands; 2 Department of Clinical Genetics, Maastricht University Medical Centre, Maastricht, 6229 ER The Netherlands; 3 Department of Toxicogenomics, GROW School for Oncology and Developmental Biology, Maastricht University Medical Centre, Maastricht, 6229 ER The Netherlands; 4 Department of Cardiology, Cardiovascular Research Institute Maastricht, CARIM School for Cardiovascular Diseases, Maastricht University Medical Centre, Maastricht, 6229 ER The Netherlands; 5 Department of Bioinformatics–BiGCaT, NUTRIM School of Nutrition Toxicology and Metabolism, Maastricht University, Maastricht, 6229 ER The Netherlands

**Keywords:** Computational biology and bioinformatics, Cardiology, Clinical genetics, Gene expression, Sequencing, Genetics, Genomics, Transcriptomics

## Abstract

Allele-specific expression (ASE) analysis detects the relative abundance of alleles at heterozygous loci as a proxy for *cis*-regulatory variation, which affects the personal transcriptome and proteome. This study describes the development and application of an ASE analysis pipeline on a unique cohort of 87 well phenotyped and RNA sequenced patients from the Maastricht Cardiomyopathy Registry with dilated cardiomyopathy (DCM), a complex genetic disorder with a remaining gap in explained heritability. Regulatory processes for which ASE is a proxy might explain this gap. We found an overrepresentation of known DCM-associated genes among the significant results across the cohort. In addition, we were able to find genes of interest that have not been associated with DCM through conventional methods such as genome-wide association or differential gene expression studies. The pipeline offers RNA sequencing data processing, individual and population level ASE analyses as well as group comparisons and several intuitive visualizations such as Manhattan plots and protein–protein interaction networks. With this pipeline, we found evidence supporting the case that *cis*-regulatory variation contributes to the phenotypic heterogeneity of DCM. Additionally, our results highlight that ASE analysis offers an additional layer to conventional genomic and transcriptomic analyses for candidate gene identification and biological insight.

## Introduction

Genome-wide association studies (GWAS) of complex phenotypes often identify non-coding variants and fail to distinguish causal variants from commonly co-inherited variants associated through linkage disequilibrium^1^. Differential gene expression (DGE) studies in turn, while offering more mechanistic insight, fail to distinguish between *cis*- and *trans*-regulatory variation^1^. In addition, allelic dosage compensation can hide mono-allelic downregulation from DGE analysis. Using RNA-sequencing data, allele-specific expression (ASE) determines the relative expression of individual alleles to find allelic imbalance caused by *cis*-acting regulatory mechanisms^1–3^. These include *cis*-acting expression and splicing quantitative trait loci (eQTLs and sQTLs), nonsense-mediated decay (NMD), X-inactivation, imprinting, and RNA interference through non-coding RNAs (ncRNA)^1–5^. The detection of allelic imbalance can be performed on a per-sample basis, which allows for the discovery of variants with low minor allele frequencies (MAF)^3^. Thus, ASE analysis enables researchers to find regulatory genomic differences regardless of total gene expression or direct variant-phenotype correlations. In complex phenotypes with low explained heritability and inter-individual differences in pathophysiology, this could contribute to the identification of causal mechanisms and therapeutic targets.

Dilated cardiomyopathy (DCM) is a complex genetic disorder characterized by dilation of the left ventricle and impaired systolic function^6,7^. Around 15–30% of DCM cases are familial, but the currently known relevant genes and variants still fail to explain 70–80% of all cases^8^. Family members of affected patients often show no evidence for familial DCM and are diagnosed with sporadic DCM^7,9,10^. Previous studies have shown eQTL enrichment for GWAS hits in two separate DCM cohorts, indicating regulatory mechanisms play a role in this phenotype^11^. The unexplained heritability and assumed regulatory mechanisms make DCM a fitting case for ASE analysis.

In this study, we have developed and applied an open-source ASE analysis pipeline in R that performs ASE analysis on the individual and population level as well as group comparisons. The aim of this pipeline is to enable the analyses of, often readily available, RNA-sequencing data in novel ways that help elucidate *cis*-regulatory processes involved in the development of complex genetic disorders. We find evidence supporting the case that *cis*-regulatory variation contributes to the phenotypic heterogeneity of DCM, discover new candidate genes, and show the benefit of performing ASE analysis in addition to GWA and differential gene expression studies.

## Results

### Allele-specific expression analysis pipeline

**Figure 1:**
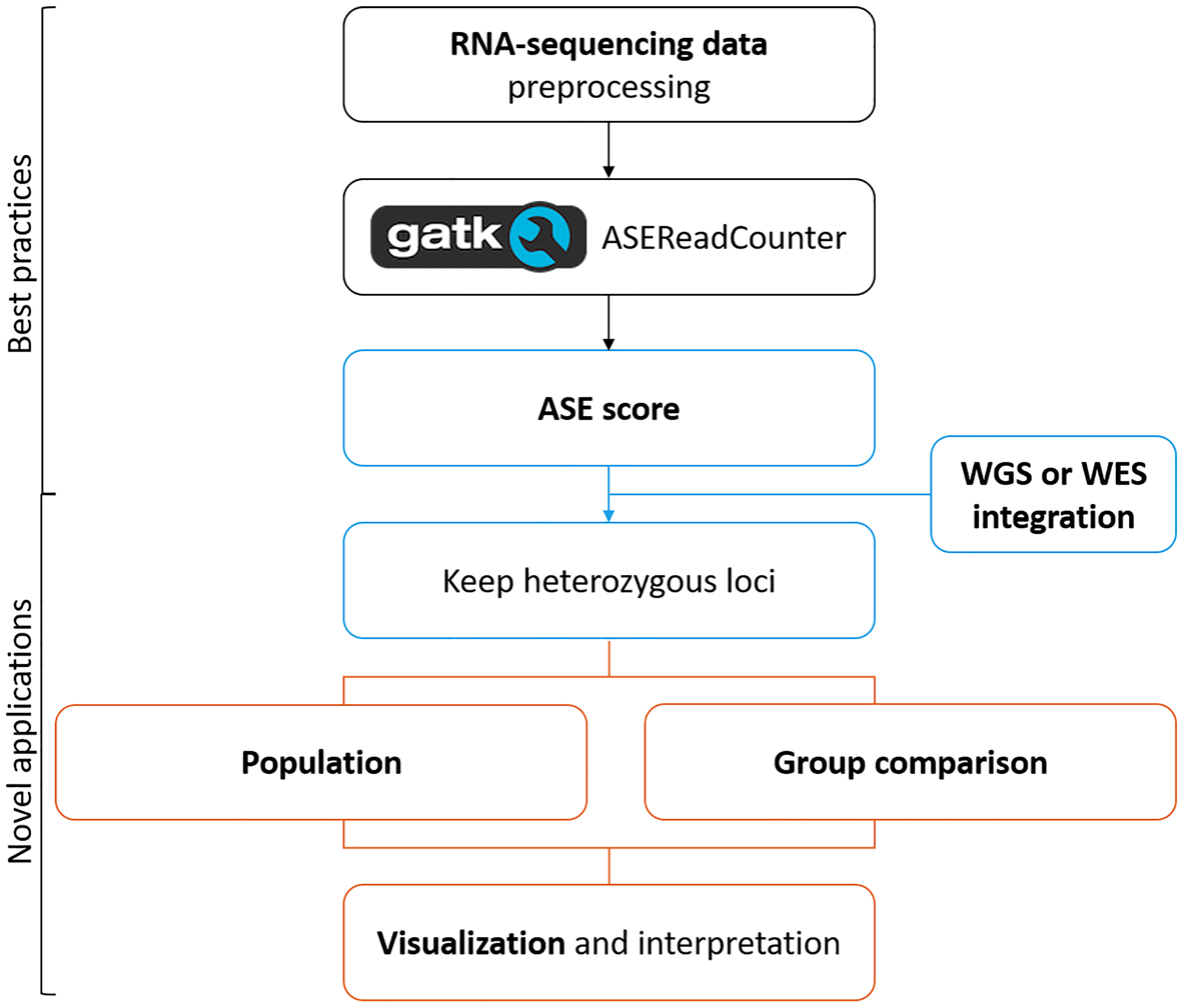
Overview of the pipeline. This analysis plan allows the inclusion of genotyping data to increase data retrieval and statistical power. The data can be evaluated on three distinct levels, with suiting visualizations for all of them in order to create interpretable results. The integration of WGS or WES data, the group comparisons, and the visualizations are novel additions to established ASE analysis pipelines.

The pipeline consists of three steps, starting with RNA sequencing data preprocessing using the Genome Analysis Toolkit (GATK), followed by the general ASE score statistics and the analyses for biological interpretation (Fig. 1) (https://github.com/macsbio/AlleleSpecificExpression). We chose to represent ASE as the absolute deviation from a heterozygous biallelic frequency of 0.5, as per the standard guidelines^3^.

### Setting the ASE score threshold to distinguish true heterozygous loci from homozygous loci

**Figure 2:**
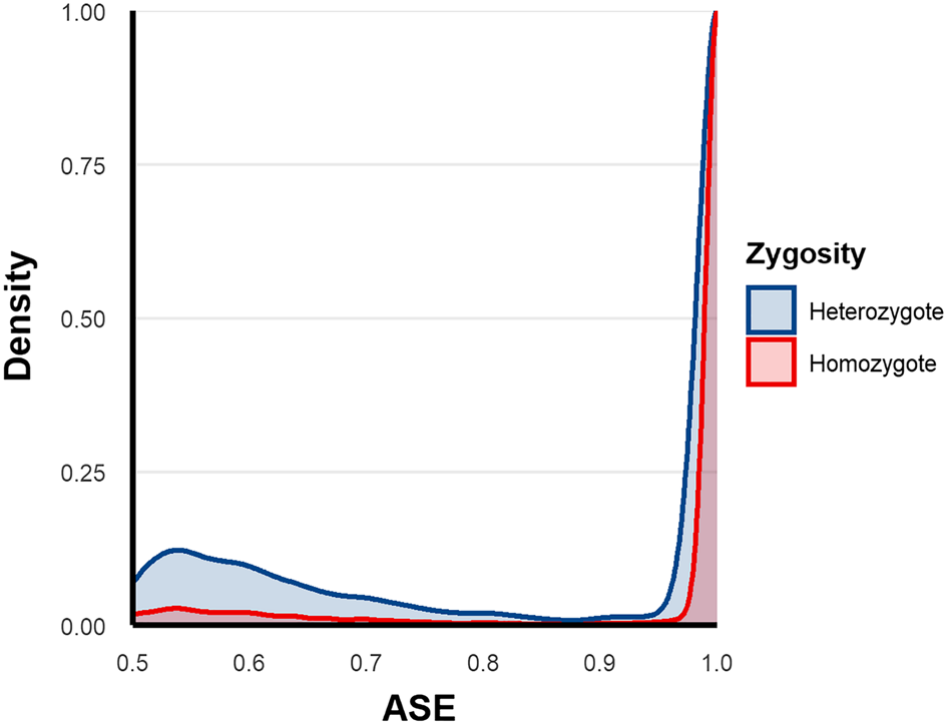
ASE density by zygosity status. This figure shows the density plots of ASE values for genotyped loci based on the zygosity status of the loci. The ASE threshold for homozygosity in non-genotyped loci is indicated with the dotted line.

The integration of genotype data allowed the determination of an ASE score threshold to distinguish between true heterozygous loci and homozygous loci with RNA sequencing artifacts. Performing Youden’s J statistic on a receiver-operating characteristic (ROC) determined an ASE score of 0.966 as the optimal threshold to distinguish between true heterozygous loci and RNA sequencing errors. The total number of heterozygous and homozygous loci was 167,329 and 719,769 respectively (Fig. 2).

### Analyzing ASE on a population level

Among all the SNPs, the total number of statistically significantly imbalanced SNPs per individual, as determined by a cutoff of q < 0.05 ranged from 210 to 8327, mean = 2093, with the percentage amongst all measured SNPs for an individual ranging between 8.9 and 81.1%, mean = 28.3% (Supplementary Materials for the full SNP-q-value list). Shared imbalance, the number of times a gene showed significant imbalance for at least one locus in each of the subjects, showed an exponentially decreasing pattern (Supplementary Fig. 1). Most genes only showed significant imbalance in one or a few of the subjects, whereas only a few genes showed imbalance in more than half of the subjects. The three genes with the highest shared imbalance showed imbalance in 79 of the samples; *ABLIM1*, *TNNT2*, and *AKAP13*, all of which have known isoforms resulting from alternative splicing^12–14^. In concordance with previous studies, the genes with at least one significantly imbalanced SNP showed significant enrichment for eQTLs, p = 6.9E^−3^, and sQTLs, p = 5.7E^−6^^10^.

### Known DCM-associated genes are more often imbalanced

**Figure 3:**
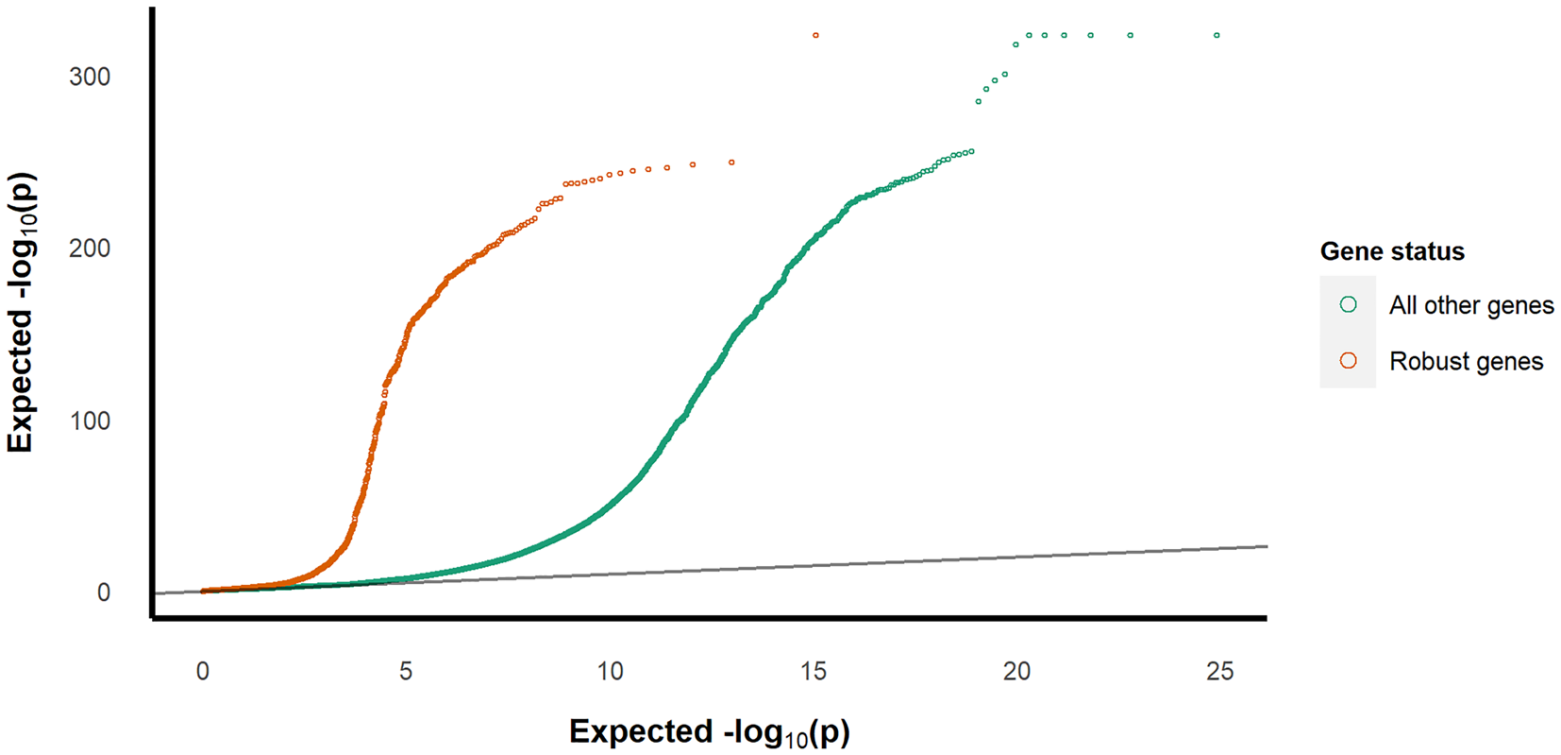
QQ-plot of p-value inflation. This figure shows the test statistic inflation for SNPs located in 12 genes with established DCM-associated variants compared to the remaining SNPs.

Since regulatory variation is known to contribute at least in part to DCM we expected to find more allelic imbalance in genes that are known to harbor variants that are associated with DCM. Thus, we compared the ASE p-value distribution for 12 genes in which confirmed DCM-associated variants most commonly occur to the p-value distribution for the rest of the dataset (Fig. 3)^15,16^. Furthermore, variants in genes classified as having a moderate, limited, or disputed link to DCM according to Hershberger et al. showed high shared imbalance across the samples and similar, albeit less pronounced, q-value inflation^17^ (Supplementary Fig. 2). When only taking the most significant SNP per gene per sample, the percentage of significant (q < 0.05) gene hits of the total dataset was 38%, while for the 12 genes with established DCM-associated variants it was 74%. The established DCM-associated genes, as described before, were more frequently observed to be significantly imbalanced in multiple samples, mean = 52 imbalanced patients (Supplementary Table 1). These findings confirm the mechanistic role of these genes in DCM.

### Differential ASE in the phenogroups comparisons

**Figure 4:**
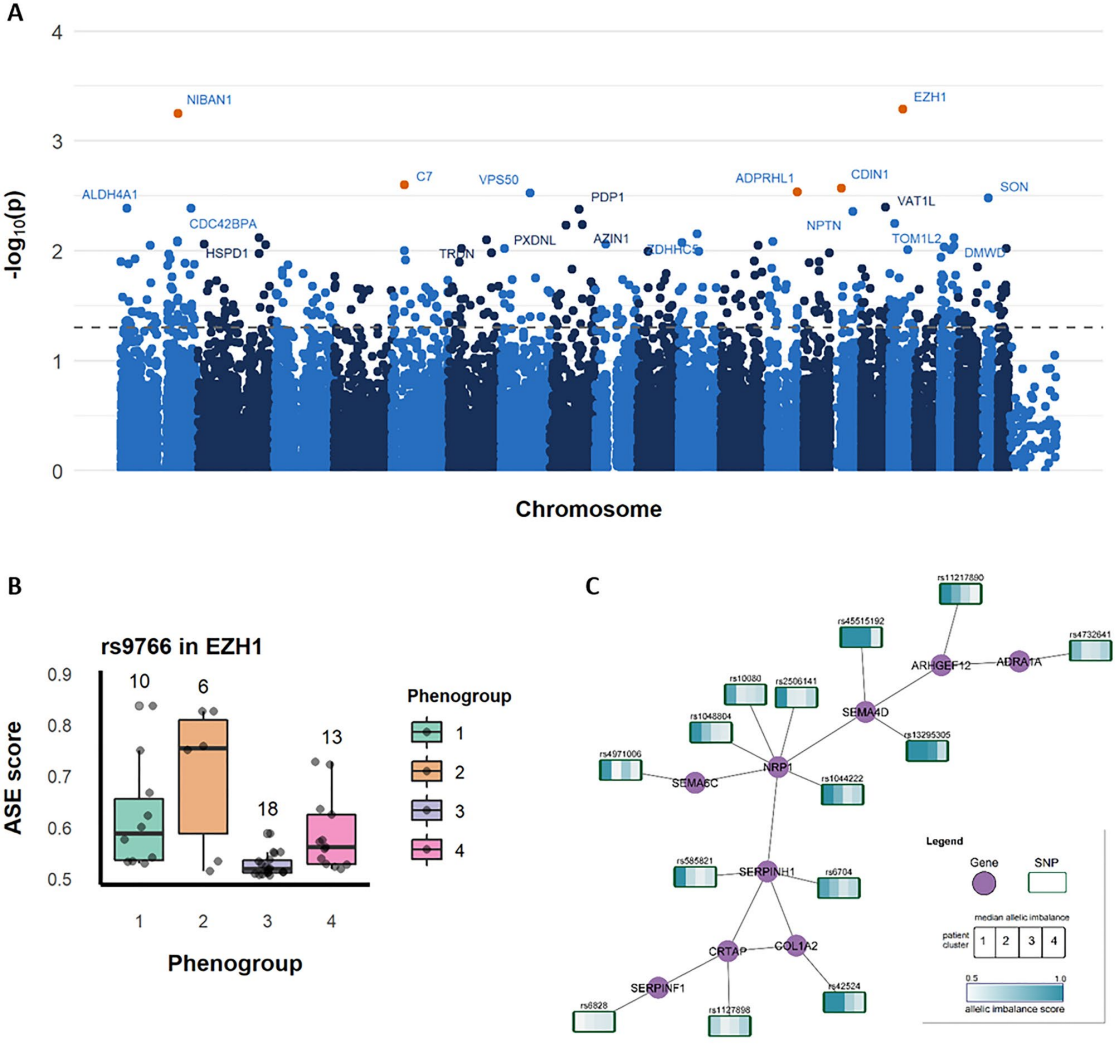
Overview of results visualizations for differential imbalance between all four phenogroups. (**A**) Manhattan plot indicating loci significantly differentially imbalanced between all four phenogroups. Note that each dot represents a SNP, annotated with the gene it is located in. (**B**) Boxplot showing the distribution of ASE scores and number of measurements by phenogroup for rs9766, located in EZH1, the most significant differentially imbalance SNP between the four phenogroups. (**C**) Subgraph of the network displaying a group of functionally related genes with median ASE scores for the corresponding SNP per phenogroup.

The DCM cohort we analyzed in this study is part of a larger set that has previously been clustered with a machine learning algorithm applied to clinical markers in order to find subsets of DCM patients with distinct phenotypical features^10^. These subsets of patients were called phenogroups and will be referred to as such from herewith (see methods for a more detailed description). In this paragraph the results and visualizations for the group comparisons are described following the Mann–Whitney U test between each phenogroup and the others as well as the Kruskal–Wallis test results between all combinations of phenogroups (see Supplementary Material for SNP-p-value lists). The Gene Ontology analyses highlighted slightly different processes when looking at genes with significant differential imbalance in one phenogroup versus the others. Metabolic processes, specifically protein metabolism and modifications, and intracellular transport processes were pronounced in phenogroup 1 (mild) and 3 (arrhythmogenic), whereas phenogroup 2 (immune) and 4 (severe) showed more pronounced effects in actin filament-based movement. Phenogroup 3 and 4 shared an enrichment for cardiac muscle contraction. Only phenogroup 1 had some enrichment for immune-related processes, specifically neutrophil activation. The topGO results are provided in the Supplementary Material. Significant differential imbalance between all four phenogroups was found for several SNPs located in genes with known cardiomyopathy links other than DCM such as posterior myocardial infarction, the top 5 hits were EZH1, NIBAN1, C7, CDIN1, and ADPRHL1 (Fig. 4A). The differential imbalance for these SNPs could be clearly visualized in boxplots (Fig. 4B, example for rs9766 in EZH1, Supplementary Material for the next four most significant hits). Genes with a SNP showing statistically differential imbalance in this analysis were visualized as a network connecting functionally related genes including their representative SNPs with median ASE scores indicated per cluster (Fig. 4C). The full network consisted of several subgraphs of varying sizes (Supplementary Material).

## Discussion

In this paper, we describe the development and application of a pipeline for ASE analysis based on standard best practices combined with novel aspects such as the incorporation of genotype data and the ability to analyze allelic imbalance on an individual, population, and group comparison level with intuitive results visualizations.

The pipeline uncovered many potential candidate genes, both known and novel in their relation to cardiomyopathy. The three genes with an imbalance in 79 of the samples, *ABLIM1*, *TNNT2*, and *AKAP13*, all have known isoforms due to alternative splicing^12–14^. Thus, these genes, as well as other commonly imbalanced genes in the dataset, might be differentially spliced and therefore showing allelic imbalance in people with DCM. This confirms prior research that showed a relation between splicing variation and DCM development^10^. Biologically, they are important for cell structure maintenance, with *TNNT2* being a troponin complex subunit and *ABLIM1* a mediator for actin-cytoplasmic interactions. *AKAP13* serves as a guanine nucleotide exchange factor for RhoA small GTPase, which is an actin regulator. Additionally, it has been shown to be essential for cardiac development in mice and has been linked to human cardiomyopathies^14,18^. Interestingly, *ABLIM1*, has been linked to DCM through *RBM24* mediated alternative splicing in knockout mice models^19^.

When looking at genes that showed differential imbalance between the autoimmunity related phenogroup 2 and the rest of the cohort, four out of the top five most significant hits were related to inflammatory processes like programmed cell death and autophagy (Supplementary Table 2)^20–23^. One of these genes, *APIP*, has been shown to carry out a cardioprotective role in the inflammatory process following myocardial infarction^20^. *TFEB* is a protein degradation promotor previously linked to autophagy and lysosomal related cardiac disorders^22^. Increased levels of *PPP1R3B*, a glycogen synthesis regulator also involved in inflammatory processes, decreases risk for myocardial infarction^24^. To our knowledge, the autophagy and intracellular protein trafficking gene *COPZ1* has not been linked to DCM previously.

For a more general overview, the Gene Ontology enrichment results on biological processes were used. While we observed little overlap with the processes attributed to the phenogroups based on a combination of gene expression and clinical data as described by Verdonschot et al. we observed additional processes that could be further investigated^11^. General cell structure and muscle fiber processes where enriched in all phenogroups, indicating that more research into the genetic regulation of these molecular processes and how these effect cardiomyocyte structure and function in DCM could be useful. Similarly, even though the 12 known DCM-associated genes showed significant imbalance in many of the samples, only *MYH7* was significantly differentially imbalanced between the four phenogroups. One explanation could be that most DCM patients have imbalances in the same core genes which would mean no differential imbalance in those genes^9^. For example, as described by Heinig et al., many DCM patients show imbalance in a wide variety of *TTN* loci^10^. The differences between phenotypic groups within DCM are more likely caused by regulatory changes in other, less disease-specific mechanisms such as inflammation and metabolism^10^. In addition, there is no reason to assume that the different phenogroups found by clustering on clinical markers are necessarily related to differential allelic imbalance. However, allelic imbalance might be a partial explanation for the heterogeneous disease progression found in DCM patients with otherwise similar or identical genetic markers. In the across phenogroups analysis, one gene that showed differential imbalance between the four groups, *ADPRHL1* as seen in Fig. 4A, codes for a protein that is key to myofibril assembly and chamber development. The gene has previously been associated with posterior myocardial infarction^25,26^. In this same analysis, the most significant differentially imbalanced hit was located in *EZH1*, a H3K27 methylation mediator involved in cardiac reprogramming^27,28^. Our results suggest that differential regulation of these genes may play a role in the etiology of different DCM subtypes.

There were several methodological considerations we encountered while designing the pipeline. The primary reason for using an ASE score from 0.5 to 1 was that this removed the distinction between reference and alternative alleles, or major and minor alleles. Since ASE is determined at the individual level, determining reference versus alternative was inapplicable. In addition, our analysis serves as a proxy for underlying regulatory variation, regardless of the direction of the imbalance. Preserving the individual alleles would therefore have added unnecessary complexity to the pipeline.

As opposed to previous ASE analysis pipelines, we decided not to aggregate the ASE scores of multiple SNPs within the same gene^3^. The rationale behind this is that some *cis*-regulatory events that cause ASE are location specific. For example, if splicing variation occurs for one of multiple exons in one allele, but not in the other allele nor for the remaining exons, ASE can only be detected for that single exon. The measured imbalance in the spliced exon would be reduced, or potentially lost, when aggregating multiple ASE measurements on the same gene. Thus, we decided to treat alleles on the same gene individually to retain a larger number of positive findings for further exploration.

Due to the threshold to classify homozygosity, all truly imbalanced heterozygous measurements with ASE above that threshold have been disregarded. This is also true for all fully imbalanced loci, where only one of the two alleles is expressed. Which could arise due to, for example, parental imprinting, and nonsense mutations. We are unaware of methodologies to circumvent this problem other than integrating genotype data for all samples.

Ultimately, this pipeline offers regulatory genetic analysis on RNA sequencing data, a commonly available genome-wide omics data. Our pipeline provides added insight into the bio-molecular etymology underlying complex regulatory genetic disorders. The visualizations, which align with the most commonly used visualizations in GWA and DGE studies, offer an intuitive understanding of the results for applications in a clinical (genetics) setting.

## Conclusion

We have shown that allele-specific expression analysis is able to pinpoint disease-relevant genes under cis-regulatory variation. Our analysis on a unique DCM cohort shows that allelic imbalances are detected in known DCM genes; furthermore, imbalances detected in novel DCM genes, while not yet experimentally or clinically linked to development of DCM, may ultimately be shown to reflect novel disease-relevant processes. Our ASE analysis pipeline can be applied at the individual and the population level and thereby play a role in research on both rare and common complex phenotypes.

## Methods

### Data description

The data set consists of a group of 87 RNA sequenced DCM patients from the Maastricht Cardiomyopathy Registry^29^. All patients were diagnosed according to World Health Organization criteria and the current European Society of Cardiology guidelines. Patients with a left ventricular ejection fraction of < 50% in the absence of obstruction of > 50% of a major coronary artery branch, pericardial diseases, congenital heart diseases, cor pulmonale, and active myocarditis were included. Unless contraindicated, patients received guideline-directed medical therapy titrated to the maximally tolerated dose and defibrillator device therapy. Endomyocardial biopsies were obtained in routine diagnostic care for each patient. All RNA was isolated from spare biopsies and sequenced using the TruSeq mRNA sample preparation kit (Illumina, San Diego, CA, USA) and the NextSeq 500 sequencing chip (Illumina, San Diego, CA, USA). All patients received genetic counseling and testing using a cardiomyopathy-associated gene panel with either single molecule molecular inversion probes or whole-exome sequencing. Whole-exome sequencing data was available for 35 patients, obtained with the Affymetrix GW6 platform (Affymetrix, Santa Carla, FL, USA). In addition, they were assigned classification labels based on a machine learning algorithm that performs clustering on DCM patients based on 28 distinct clinical features^11^. This resulted in four phenotypically distinct patient clusters (phenogroups) with increasing disease severity. Phenogroup 1 was mainly characterized by a moderate ejection fraction with low creatine levels and overall mild disease symptoms, phenogroup 2 by high creatine levels and an overrepresentation of auto-immune disease diagnoses, phenogroup 3 by the presence of atrial fibrillation, and phenogroup 4 by a low ejection fraction and other end-stage symptoms. All patients gave written informed consent before inclusion and the relevant guidelines and regulations were strictly adhered to^29^.

### Data preparation

#### RNA sequencing data processing

RNA sequencing data was processed using the Genome Analysis Toolkit (GATK, version 3.8.1) for variant calling and read counting. All following steps were performed in R version 4.0.2^30^. The fraction of reference and alternative allele reads was calculated for all loci. As a means of standardization, the ASE scores were set as the absolute deviation of the allelic read fraction from 0.5 to 1.0 as commonly done by others (Eq. 1) (ASE score calculation)^2,3^.

1 $$ASE \; score=\left|\left(\frac{{Read \;count}_{reference}}{{Read \;count}_{total}}\right)-0.5\right|+0.5$$

### Distinguishing high imbalance from artifacts

The subset of samples with available whole-exome sequencing data was used to determine a homozygosity threshold based on the ASE score since the true zygosity for these samples was known. A receiver operating characteristic (ROC) analysis was performed using a model of zygosity (1 for homozygosity, 0 for heterozygosity) as a function of the ASE score^31^. Next, Youden’s J statistic was applied to find the threshold that maximizes the distance to the ROC diagonal^31^. Due to unequal sample sizes between homozygous and heterozygous loci, resampling was applied. The mean ASE score threshold was then used to filter out likely homozygous loci within the non-genotyped subset.

### Testing for statistical significance

Statistical tests were performed on the ASE scores on a per sample basis as well as between phenogroups. Within-sample ASE significance was determined using a binomial test, where the expected probability of finding a certain allele in the total read count at a particular locus was set to be the median of all median ASE values per sample (0.647). Multiple testing correction was performed for the binomial test results using the Benjamini–Hochberg method^32^. For the group comparisons, a Wilcoxon rank-sum test was applied to calculate the statistical significance of differential imbalance between pairs of phenogroups. To investigate ASE variation between all four phenogroups, we performed the Kruskal–Wallis Rank-Sum Test. Non-parametric tests were chosen due to the non-normality of the ASE scores as well as the sparse measurements per SNP across the samples.

### Biological interpretation

All SNP identifiers in the output results were mapped to Ensembl gene identifiers (ENSG IDs) and HUGO Gene Name Committee (HGNC) symbols^33^. For genes with multiple ASE measurements, the SNP with the lowest p-value was taken to represent the gene for that individual, since several of the potential underlying mechanisms of ASE, such as splicing and transcript truncation (followed by NMD), are position-specific and cannot be accurately aggregated with imbalances in other exons^4,10^. Ensembl Gene Ontology data and SNP-to-gene mapping were subsequently used to perform biological interpretation. The R-package topGO was used to perform Gene Ontology enrichment analysis using the parent–child for all significant gene hits from each analysis^34,35^. A gene was considered statistically significant if the lowest q-value for an ASE event within that gene was < 0.05. Genes with a SNP showing statistically significant differential imbalance between the four phenogroups were visualized as a network connecting functionally related genes including their representative SNPs with median ASE scores indicated per cluster. This network was created by taking the related genes of differentially imbalanced SNPs and, after translating gene identifiers to protein identifiers, using the STRING database to find protein–protein interactions with a cut-off of 0.9^36^.

### Ethics approval and consent to participate

All patients gave written informed consent before inclusion^29^. An independent Medical Ethics Committee of the Maastricht University Medical Center (MUMC+) has approved this registry.

## Supplementary Information

Supplementary Legends.

Supplementary Information 2.

## Data Availability

RNA-sequencing data are available through gene expression omnibus (GEO) under accession number GSE146621. The clinical data that support the findings of this study are available from the corresponding author on reasonable request (https://www.maastrichtheartandvascularcenter.com/research/collaborate). All scripts created and used in Bash and R are available on GitHub (https://github.com/macsbio/AlleleSpecificExpression).

## References

[1] Rao X, Thapa KS, Chen AB, Lin H, Gao H, Reiter JL (2019). Allele-specific expression and high-throughput reporter assay reveal functional genetic variants associated with alcohol use disorders. Mol. Psychiatry..

[2] Castel SE, Levy-Moonshine A, Mohammadi P, Banks E, Lappalainen T (2015). Tools and best practices for data processing in allelic expression analysis. Genome Biol..

[3] Fan J, Hu J, Xue C, Zhang H, Susztak K, Reilly MP (2020). ASEP: Gene-based detection of allele-specific expression across individuals in a population by RNA sequencing. PLoS Genet..

[4] Demirdjian L, Xu Y, Bahrami-Samani E, Pan Y, Stein S, Xie Z (2020). Detecting allele-specific alternative splicing from population-scale RNA-Seq data. Am. J. Hum. Genet..

[5] Langmyhr M, Henriksen SP, Cappelletti C, van de Berg WDJ, Pihlstrom L, Toft M (2021). Allele-specific expression of Parkinson's disease susceptibility genes in human brain. Sci. Rep..

[6] Schultheiss HP, Fairweather D, Caforio ALP, Escher F, Hershberger RE, Lipshultz SE (2019). Dilated cardiomyopathy. Nat. Rev. Dis. Primers..

[7] Fatkin D, Seidman CE, Seidman JG (2014). Genetics and disease of ventricular muscle. Cold Spring Harb. Perspect. Med..

[8] Petretta M, Pirozzi F, Sasso L, Paglia A, Bonaduce D (2011). Review and metaanalysis of the frequency of familial dilated cardiomyopathy. Am. J. Cardiol..

[9] Rosenbaum AN, Agre KE, Pereira NL (2020). Genetics of dilated cardiomyopathy: Practical implications for heart failure management. Nat. Rev. Cardiol..

[10] Heinig M, Adriaens ME, Schafer S, van Deutekom HWM, Lodder EM, Ware JS (2017). Natural genetic variation of the cardiac transcriptome in non-diseased donors and patients with dilated cardiomyopathy. Genome Biol..

[11] Verdonschot JAJ, Merlo M, Dominguez F, Wang P, Henkens M, Adriaens ME (2021). Phenotypic clustering of dilated cardiomyopathy patients highlights important pathophysiological differences. Eur. Heart J..

[12] Boeckel JN, Mobius-Winkler M, Muller M, Rebs S, Eger N, Schoppe L (2022). SLM2 is a novel cardiac splicing factor involved in heart failure due to dilated cardiomyopathy. Genomics Proteomics Bioinform..

[13] Yamamoto T, Miura A, Itoh K, Takeshima Y, Nishio H (2019). RNA sequencing reveals abnormal LDB3 splicing in sudden cardiac death. Forensic Sci. Int..

[14] Mayers CM, Wadell J, McLean K, Venere M, Malik M, Shibata T (2010). The Rho guanine nucleotide exchange factor AKAP13 (BRX) is essential for cardiac development in mice. J. Biol. Chem..

[15] Mazzarotto F, Tayal U, Buchan RJ, Midwinter W, Wilk A, Whiffin N (2020). Reevaluating the genetic contribution of monogenic dilated cardiomyopathy. Circulation.

[16] Stroeks S, Hellebrekers D, Claes GRF, Tayal U, Krapels IPC, Vanhoutte EK (2021). Clinical impact of re-evaluating genes and variants implicated in dilated cardiomyopathy. Genet. Med..

[17] Jordan E, Peterson L, Ai T, Asatryan B, Bronicki L, Brown E (2021). Evidence-based assessment of genes in dilated cardiomyopathy. Circulation.

[18] Johnson KR, Nicodemus-Johnson J, Spindler MJ, Carnegie GK (2015). Genome-wide gene expression analysis shows AKAP13-mediated PKD1 signaling regulates the transcriptional response to cardiac hypertrophy. PLoS ONE.

[19] Liu J, Kong X, Zhang M, Yang X, Xu X (2019). RNA binding protein 24 deletion disrupts global alternative splicing and causes dilated cardiomyopathy. Protein Cell..

[20] Lim B, Jung K, Gwon Y, Oh JG, Roh JI, Hong SH (2019). Cardioprotective role of APIP in myocardial infarction through ADORA2B. Cell Death Dis..

[21] Di Marco T, Bianchi F, Sfondrini L, Todoerti K, Bongarzone I, Maffioli EM (2020). COPZ1 depletion in thyroid tumor cells triggers type I IFN response and immunogenic cell death. Cancer Lett..

[22] Wundersitz S, Pablo Tortola C, Schmidt S, Oliveira Vidal R, Kny M, Hahn A (2022). The transcription factor EB (TFEB) sensitizes the heart to chronic pressure overload. Int. J. Mol. Sci..

[23] Noordam R, Oudt CH, Bos MM, Smit RAJ, van Heemst D (2018). High-sensitivity C-reactive protein, low-grade systemic inflammation and type 2 diabetes mellitus: A two-sample Mendelian randomization study. Nutr. Metab. Cardiovasc. Dis..

[24] Kahali B, Chen Y, Feitosa MF, Bielak LF, O'Connell JR, Musani SK (2021). A noncoding variant near PPP1R3B promotes liver glycogen storage and MetS, but protects against myocardial infarction. J. Clin. Endocrinol. Metab..

[25] Smith SJ, Towers N, Saldanha JW, Shang CA, Mahmood SR, Taylor WR (2016). The cardiac-restricted protein ADP-ribosylhydrolase-like 1 is essential for heart chamber outgrowth and acts on muscle actin filament assembly. Dev. Biol..

[26] Smith SJ, Towers N, Demetriou K, Mohun TJ (2020). Defective heart chamber growth and myofibrillogenesis after knockout of adprhl1 gene function by targeted disruption of the ancestral catalytic active site. PLoS ONE.

[27] Dal-Pra S, Hodgkinson CP, Mirotsou M, Kirste I, Dzau VJ (2017). Demethylation of H3K27 is essential for the induction of direct cardiac reprogramming by miR combo. Circ. Res..

[28] Kook H, Seo SB, Jain R (2017). EZ switch from EZH2 to EZH1: Histone methylation opens a window of cardiac regeneration. Circ. Res..

[29] Henkens M, Weerts J, Verdonschot JAJ, Raafs AG, Stroeks S, Sikking MA (2022). Improving diagnosis and risk stratification across the ejection fraction spectrum: The Maastricht Cardiomyopathy registry. ESC Heart Fail..

[30] R Development Core Team (2021). R: A Language and Environment for Statistical Computing.

[31] Robin X, Turck N, Hainard A, Tiberti N, Lisacek F, Sanchez JC (2011). pROC: An open-source package for R and S+ to analyze and compare ROC curves. BMC Bioinform..

[32] Storey JD, Tibshirani R (2003). Statistical significance for genomewide studies. Proc. Natl. Acad. Sci. USA..

[33] Durinck S, Spellman PT, Birney E, Huber W (2009). Mapping identifiers for the integration of genomic datasets with the R/Bioconductor package biomaRt. Nat. Protoc..

[34] Grossmann S, Bauer S, Robinson PN, Vingron M (2007). Improved detection of overrepresentation of Gene-Ontology annotations with parent child analysis. Bioinformatics.

[35] Alexa, A. & Rahnenführer, J. *topGO: Enrichment Analysis for Gene Ontology.* (2021).

[36] Szklarczyk D, Gable AL, Lyon D, Junge A, Wyder S, Huerta-Cepas J (2019). STRING v11: Protein–protein association networks with increased coverage, supporting functional discovery in genome-wide experimental datasets. Nucleic Acids Res..

