# Supplementary material for: Allele-specific expression analysis for complex genetic phenotypes applied to a unique dilated cardiomyopathy cohort
Source: Sci Rep. 2023 Jan 11;13:564. doi: 10.1038/s41598-023-27591-7 (PMC9834222; doi:10.1038/s41598-023-27591-7)
Supplement: Supplementary file 1 — Supplementary Legends. [file 41598_2023_27591_MOESM1_ESM.docx]

**Allele-specific Expression Analysis for Complex Genetic Phenotypes Applied to A Unique Dilated Cardiomyopathy Cohort**

**Author Names**

Daan van Beek^1^ d.beek@maastrichtuniversity.nl

Job Verdonschot^2^ job.verdonschot@mumc.nl

Kasper Derks^2^ kasper.derks@mumc.nl

Han Brunner^2^ han.brunner@mumc.nl

Theo M. de Kok^1, 3^ t.dekok@maastrichtuniversity.nl

Ilja C. W. Arts^1^ ilja.arts@maastrichtuniversity.nl

Stephane Heymans^4^ s.heymans@maastrichtuniversity.nl

Martina Kutmon^1, 5, #^ martina.kutmon@maastrichtuniversity.nl

Michiel Adriaens^1, #,*^ michiel.adriaens@maastrichtuniversity.nl

^1^ Maastricht Centre for Systems Biology (MaCSBio), Maastricht University, Maastricht, 6229 EN, Netherlands

^2^ Department of Clinical Genetics, Maastricht University Medical Centre, Maastricht, 6229 ER, Netherlandss

^3^ Department of Toxicogenomics, GROW School for Oncology and Developmental Biology, Maastricht University Medical Centre, Maastricht, 6229 ER, Netherlands

^4^ Department of Cardiology, Cardiovascular Research Institute Maastricht, CARIM School for Cardiovascular Diseases, Maastricht University Medical Centre, Maastricht, 6229 ER, Netherlands

^5^ Department of Bioinformatics – BiGCaT, NUTRIM School of Nutrition Toxicology and Metabolism, Maastricht University, Maastricht, 6229 ER, Netherlands

^*^ Corresponding author, michiel.adriaens@maastrichtuniversity.nl

^#^ Equal contribution

**DESCRIPTION OF FILES**

Group_2vs1_results: Manhattan plot for phenogroup 1 versus 2 comparison, boxplot for ASE distribution of most significant imbalanced locus in this analysis, topGO results from this analysis.

Group_3vs1_results: Manhattan plot for phenogroup 1 versus 3 comparison, boxplot for ASE distribution of most significant imbalanced locus in this analysis, topGO results from this analysis.

Group_3vs2_results: Manhattan plot for phenogroup 2 versus 3 comparison, boxplot for ASE distribution of most significant imbalanced locus in this analysis, topGO results from this analysis.

Group_4vs1_results: Manhattan plot for phenogroup 1 versus 4 comparison, boxplot for ASE distribution of most significant imbalanced locus in this analysis, topGO results from this analysis.

Group_4vs2_results: Manhattan plot for phenogroup 2 versus 4 comparison, boxplot for ASE distribution of most significant imbalanced locus in this analysis, topGO results from this analysis.

Group_4vs3_results: Manhattan plot for phenogroup 3 versus 4 comparison, boxplot for ASE distribution of most significant imbalanced locus in this analysis, topGO results from this analysis.

Boxplot_rs14023_across

Boxplot_rs3784678_across

Boxplot_rs9603837_across

Boxplot_rs10473230_across: Boxplots of the four most significant differentially imbalanced loci in the across phenogroups analysis.

Cluster_1_topGO_33perc

Cluster_1_topGO_total: topGO results for all genes with significantly imbalanced loci in samples from phenogroup 1, and for those genes with imbalanced loci in at least a third of the samples from phenogroup 1 (and so forth for the other 3 phenogroups).

results_1vAll: SNP-Pvalue table of the comparison between phenogroup 1 and the others (and so forth for other phenogroups).

results_across_groups: SNP-Pvalue table of the across phenogroups analysis.

results_total_cohort: Results of the first, individual-level, ASE analysis showing SNP-Pvalue per sample ID (SID). This allows those interested to see which loci from which genes were (significantly) imbalanced in which samples.

Supplementary_Figure1: Histogram showing the (y) amount of times a gene has an imbalanced loci in (x) the number of samples (e.g. there are around 200 genes with an imbalanced loci in around 20 of the samples).

Supplementary_Figure2: QQ-plot for SNPs located in the genes with a moderate to limited link to DCM.

Supplementary_Table1: Showing amount of samples with an imbalanced locus in the robust DCM genes.

topGO_total: topGO results from all genes with loci with a significant imbalance in any sample.

topGO_33perc: topGO results from all genes with loci with a significant imbalance in at least a third of all samples.

topGO_cluster1_vsAll: topGO results from all genes with loci with a significant differential imbalance in the analysis of phenogroup 1 versus the rest (and so forth for the other phenogroups).

**Supplementary Table 2** (topGO_cluster2_vsAll, first sheet (Biological Process)) **Table Legend**: This table shows the topGO results from all genes with loci with a significant differential imbalance in the analysis of phenogroup 2 versus the rest. Columns from left to right describe (1) the Gene Ontology ID, (2) the name of the process, (3) the number of total hits in a process, (4) the number of hits that are significant, (5) the expected number of significant hits, and (6) the pvalue.
