# Supplementary figures and images for: Allele-specific expression analysis for complex genetic phenotypes applied to a unique dilated cardiomyopathy cohort
Source: Sci Rep. 2023 Jan 11;13:564. doi: 10.1038/s41598-023-27591-7 (PMC9834222; doi:10.1038/s41598-023-27591-7)

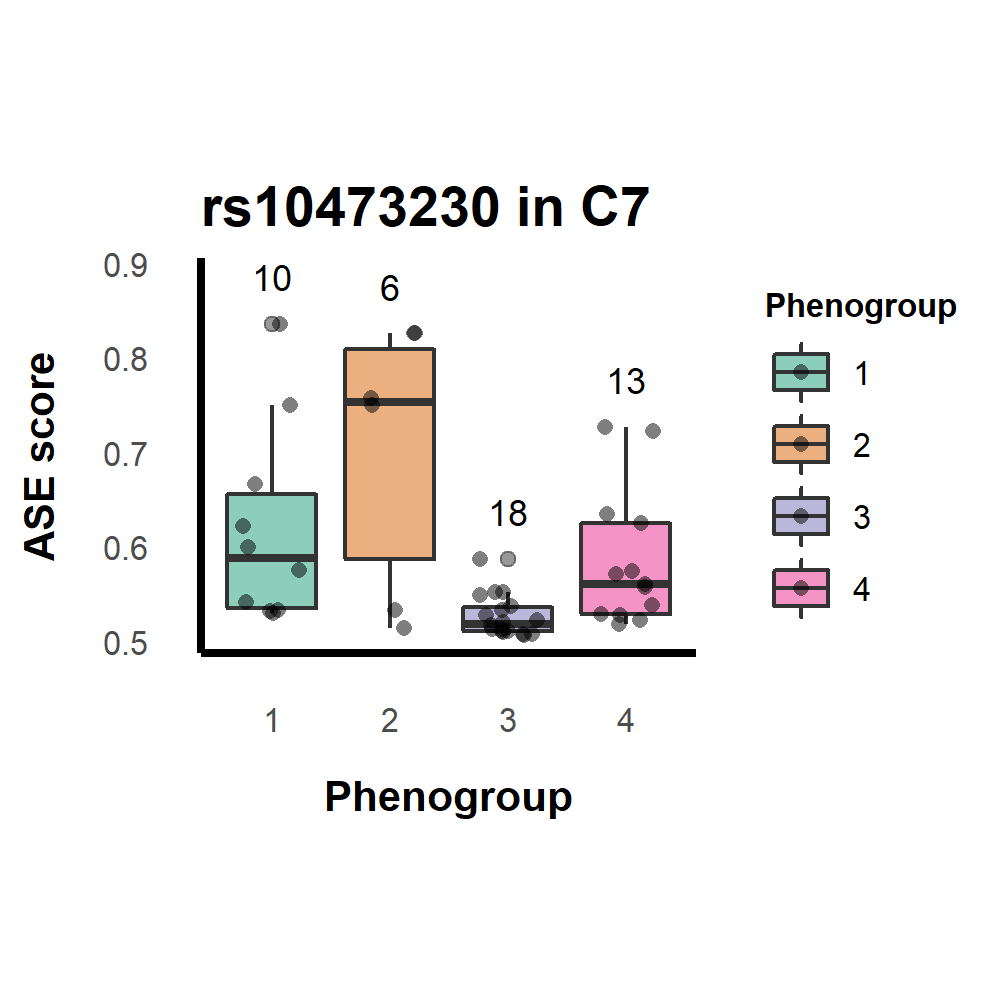

Supplement: Supplementary file 2 — Supplementary Information 2. [file 41598_2023_27591_MOESM2_ESM.zip › Boxplot_rs10473230_across.tif]

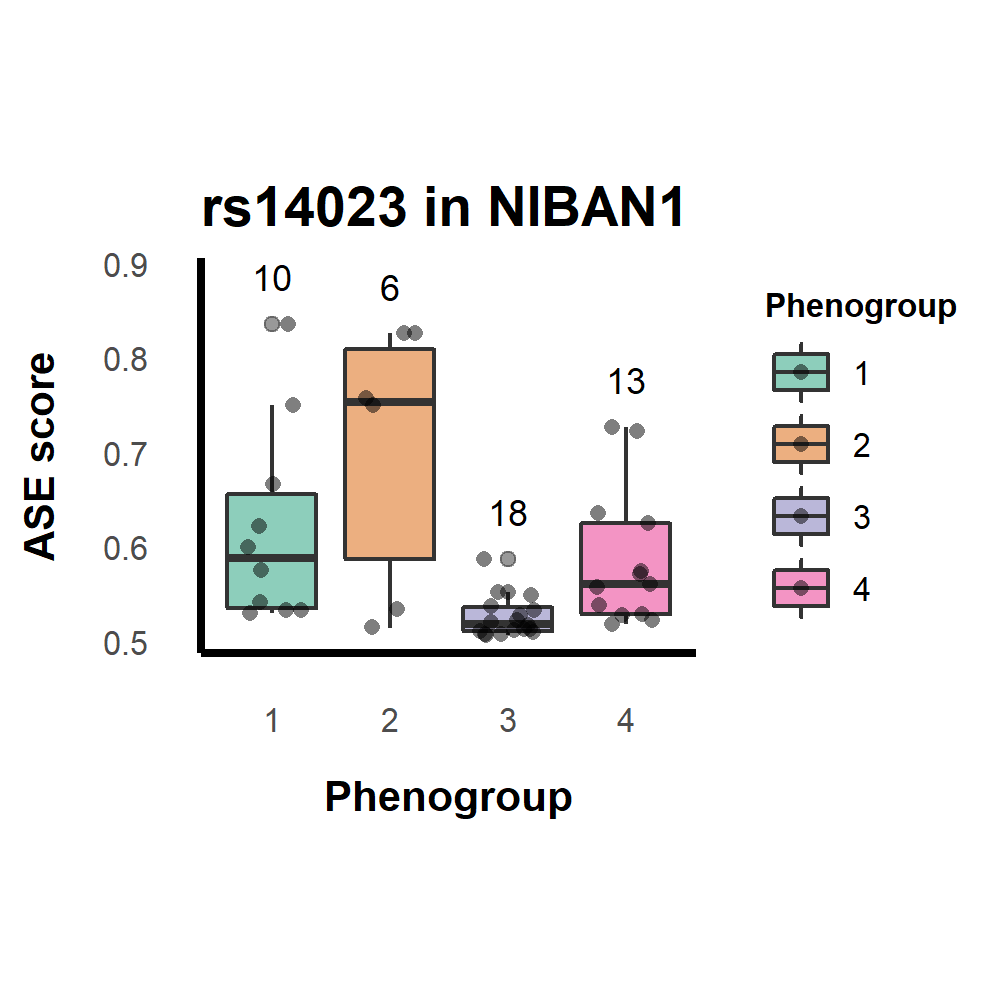

Supplement: Supplementary file 2 — Supplementary Information 2. [file 41598_2023_27591_MOESM2_ESM.zip › Boxplot_rs14023_across.tif]

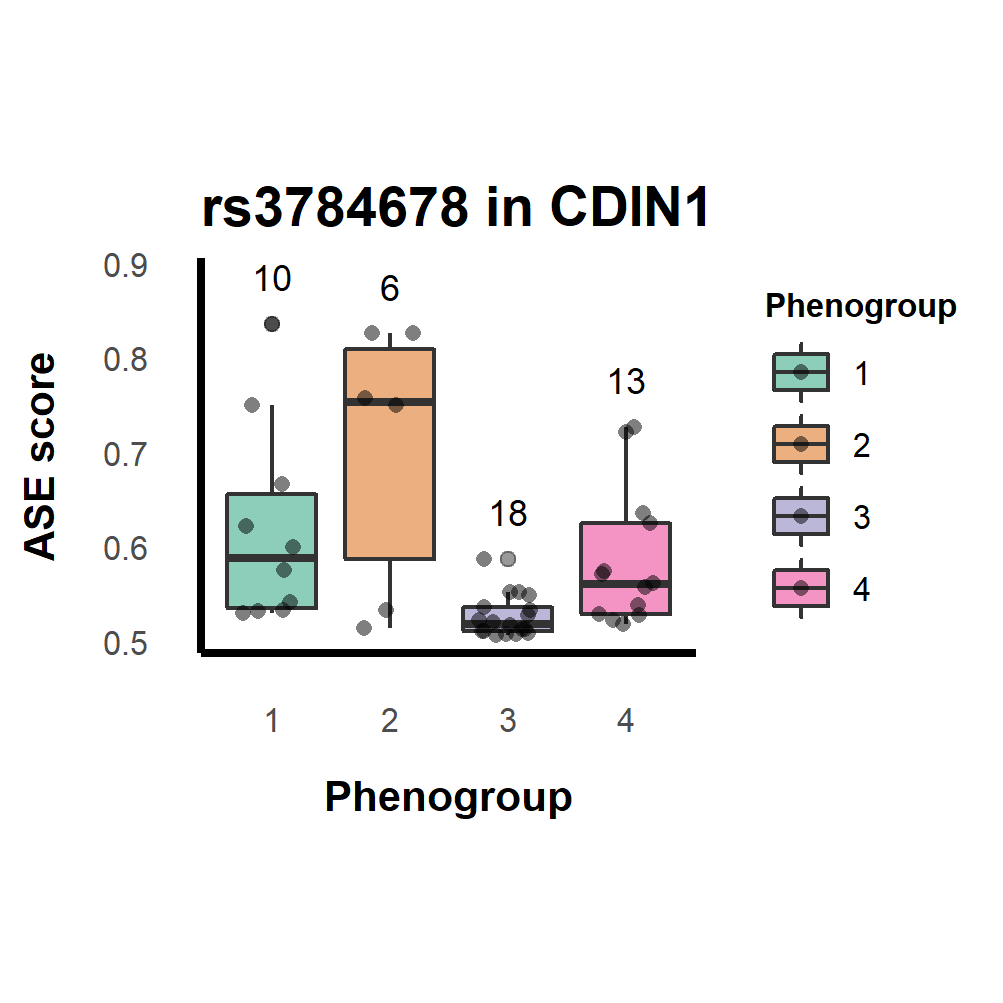

Supplement: Supplementary file 2 — Supplementary Information 2. [file 41598_2023_27591_MOESM2_ESM.zip › Boxplot_rs3784678_across.tif]

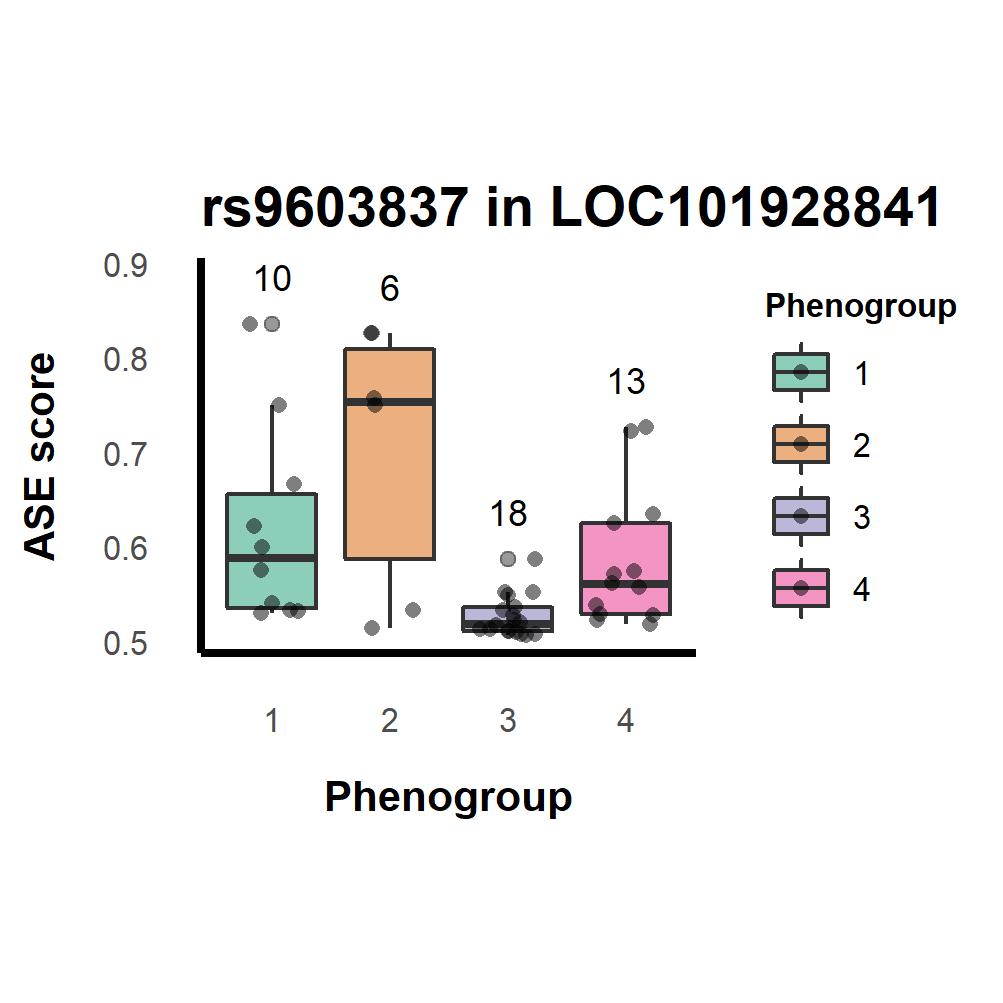

Supplement: Supplementary file 2 — Supplementary Information 2. [file 41598_2023_27591_MOESM2_ESM.zip › Boxplot_rs9603837_across.tif]

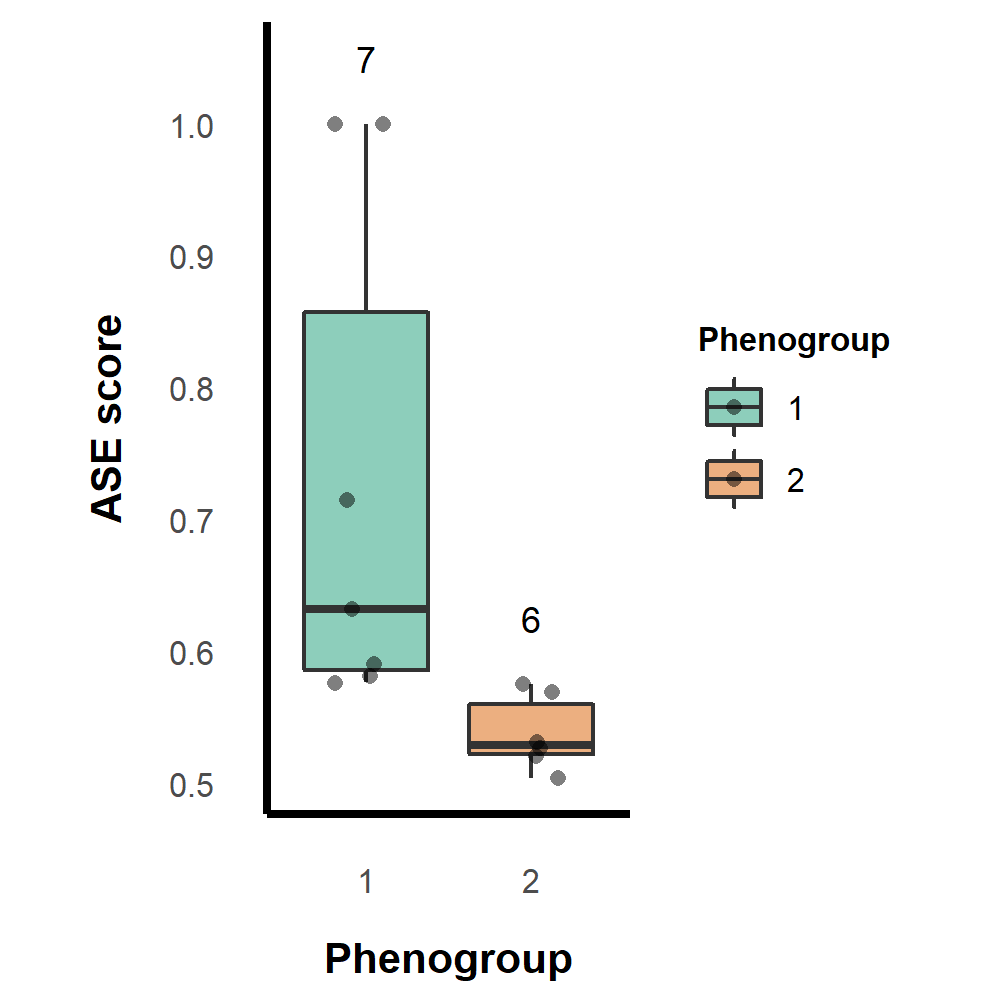

Supplement: Supplementary file 2 — Supplementary Information 2. [file 41598_2023_27591_MOESM2_ESM.zip › Group_2vs1_results/Boxplot_rs9766_across.tif]

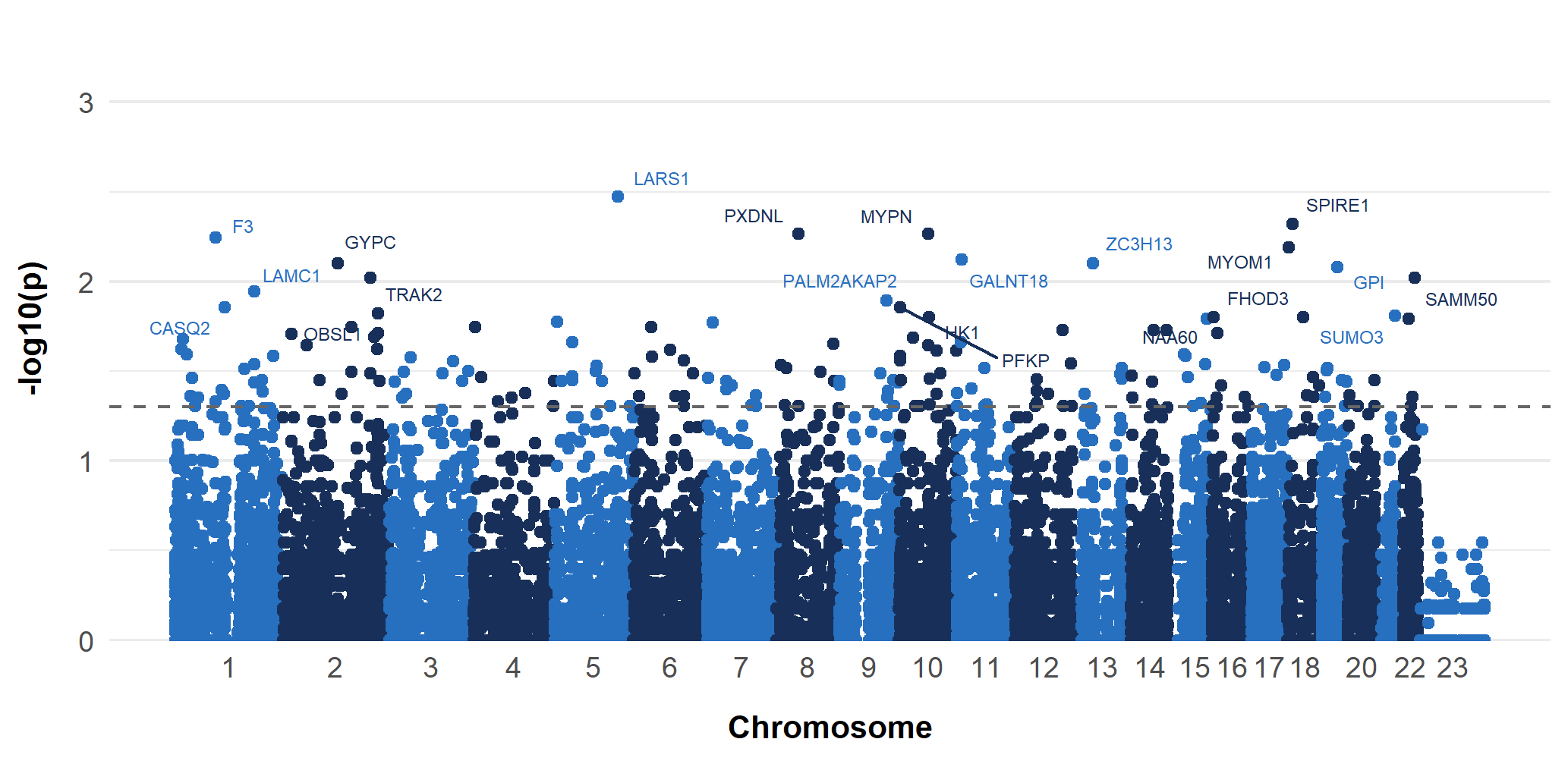

Supplement: Supplementary file 2 — Supplementary Information 2. [file 41598_2023_27591_MOESM2_ESM.zip › Group_2vs1_results/Group_2vs1_Manhattan.tif]

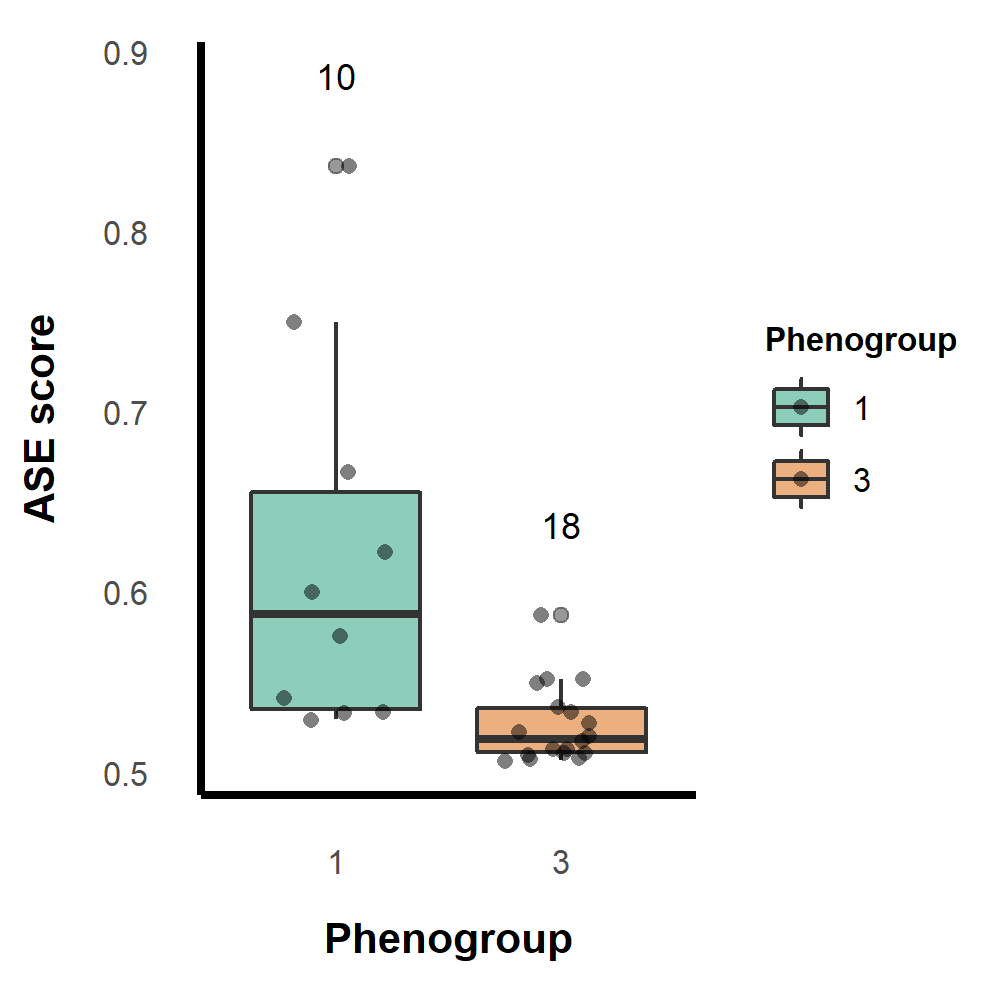

Supplement: Supplementary file 2 — Supplementary Information 2. [file 41598_2023_27591_MOESM2_ESM.zip › Group_3vs1_results/Boxplot_rs9766_across.tif]

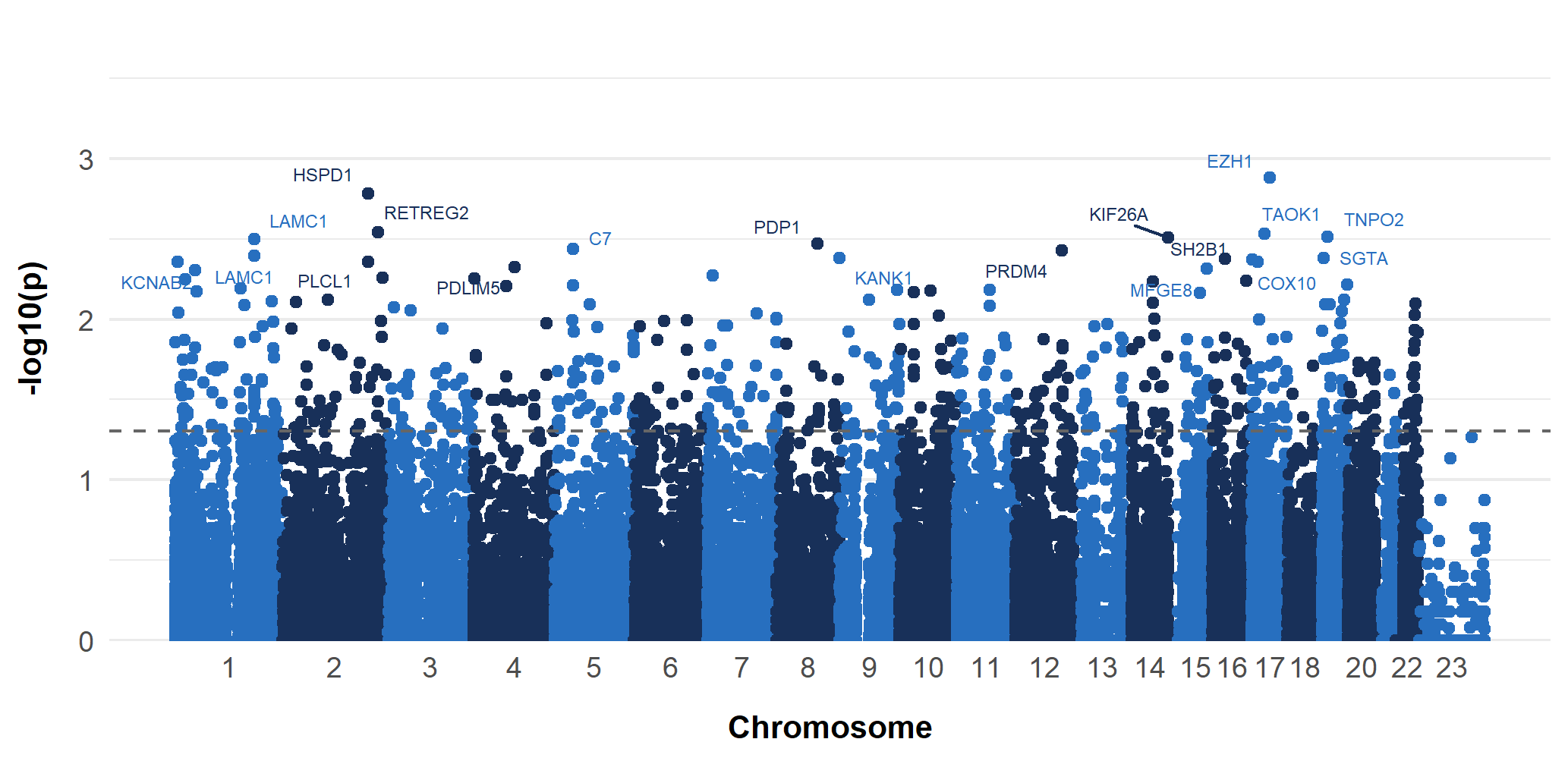

Supplement: Supplementary file 2 — Supplementary Information 2. [file 41598_2023_27591_MOESM2_ESM.zip › Group_3vs1_results/Group_3vs1_Manhattan.tif]

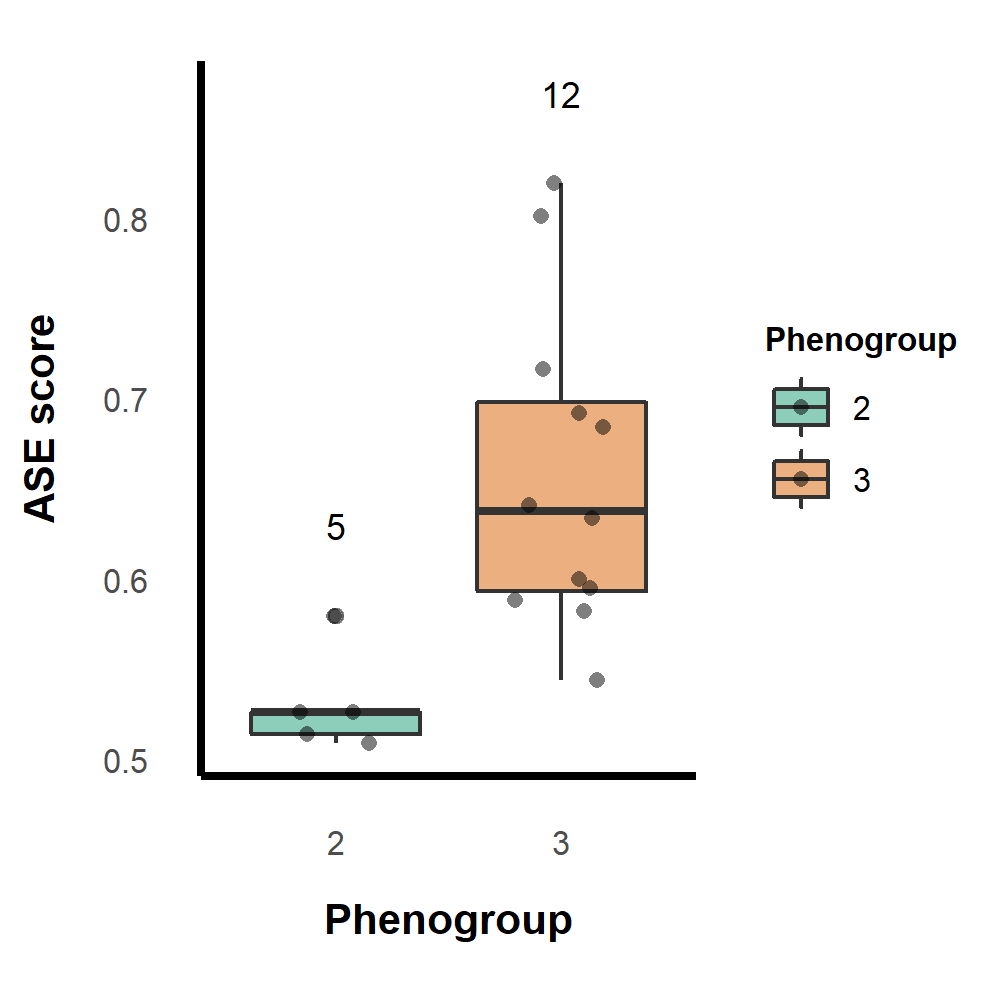

Supplement: Supplementary file 2 — Supplementary Information 2. [file 41598_2023_27591_MOESM2_ESM.zip › Group_3vs2_results/Boxplot_rs9766_across.tif]

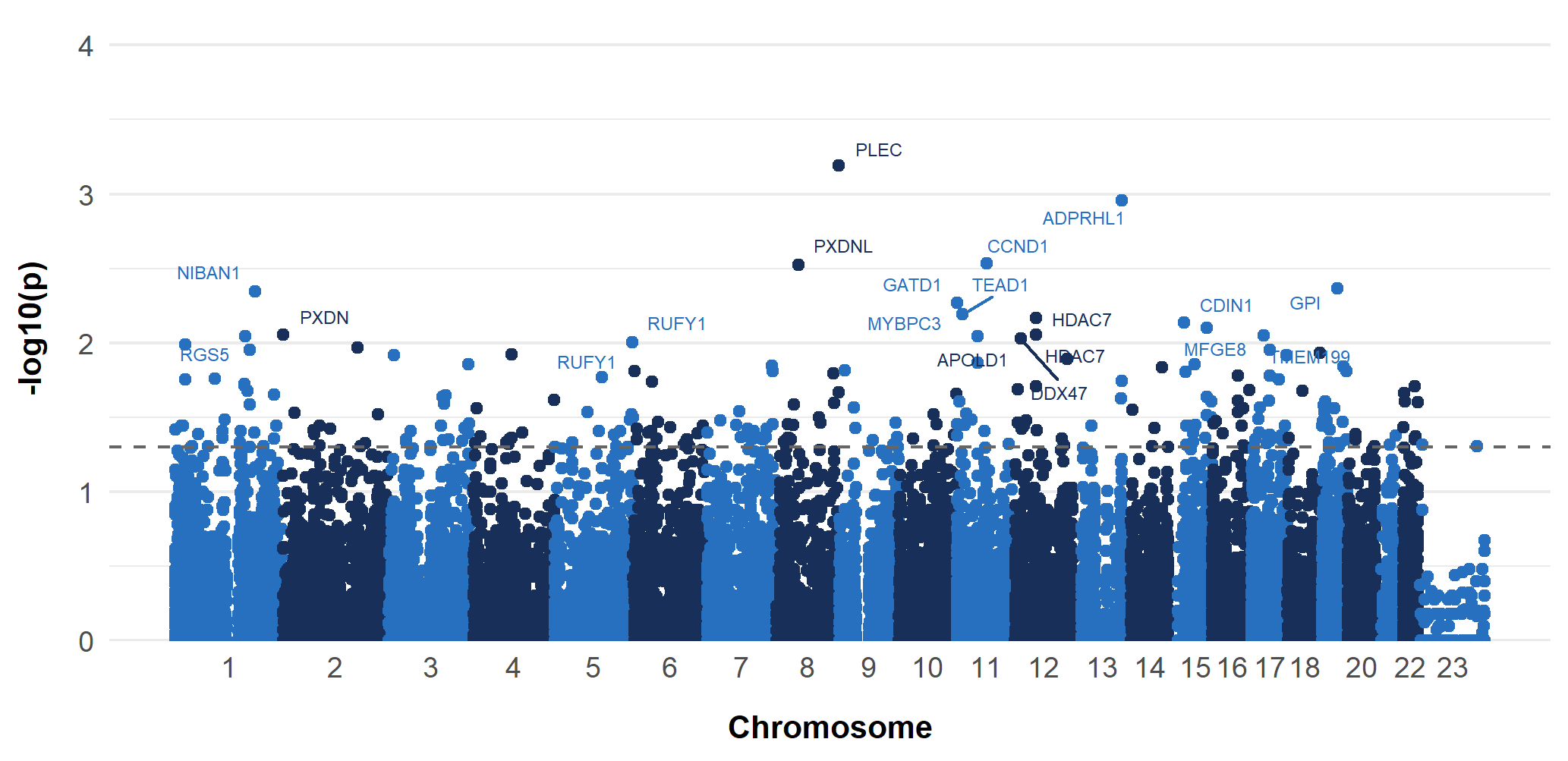

Supplement: Supplementary file 2 — Supplementary Information 2. [file 41598_2023_27591_MOESM2_ESM.zip › Group_3vs2_results/Group_3vs2_Manhattan.tif]

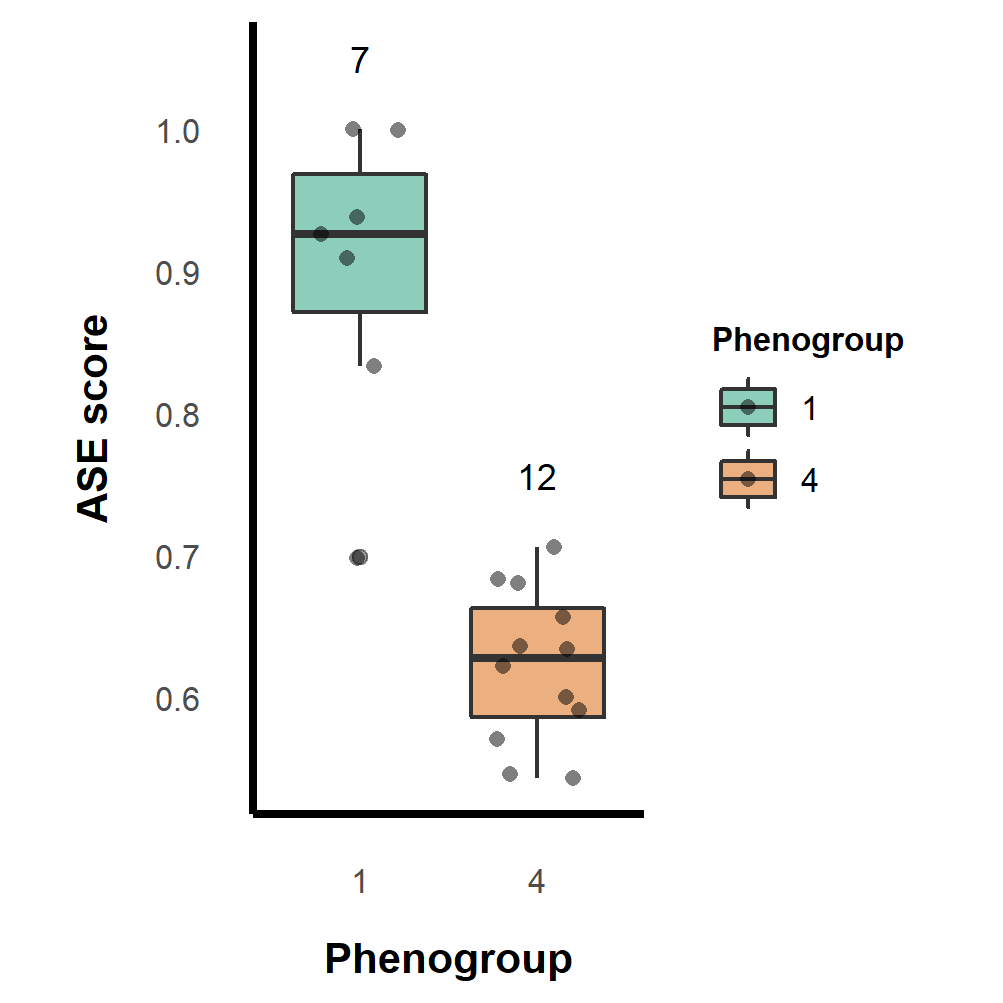

Supplement: Supplementary file 2 — Supplementary Information 2. [file 41598_2023_27591_MOESM2_ESM.zip › Group_4vs1_results/Boxplot_rs9766_across.tif]

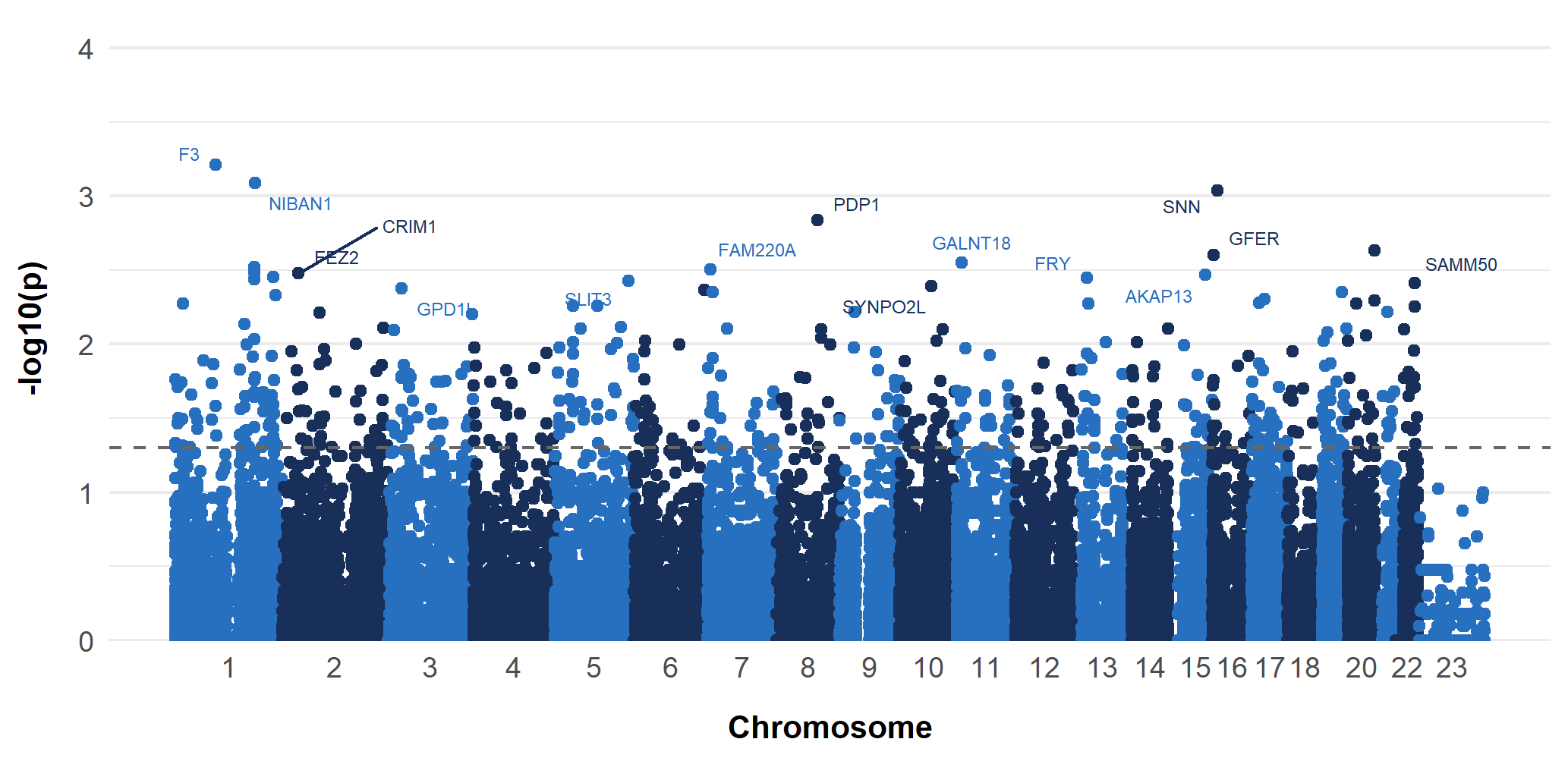

Supplement: Supplementary file 2 — Supplementary Information 2. [file 41598_2023_27591_MOESM2_ESM.zip › Group_4vs1_results/Group_4vs1_Manhattan.tif]

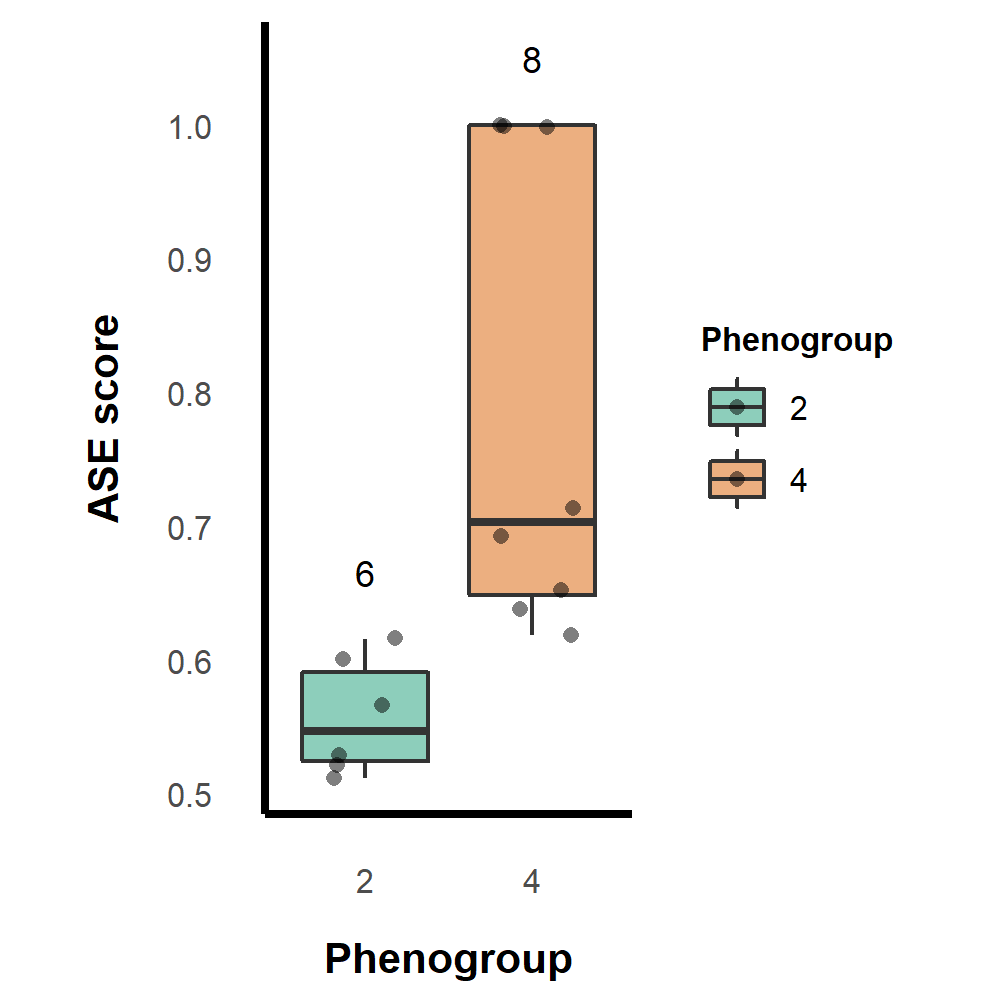

Supplement: Supplementary file 2 — Supplementary Information 2. [file 41598_2023_27591_MOESM2_ESM.zip › Group_4vs2_results/Boxplot_rs9766_across.tif]

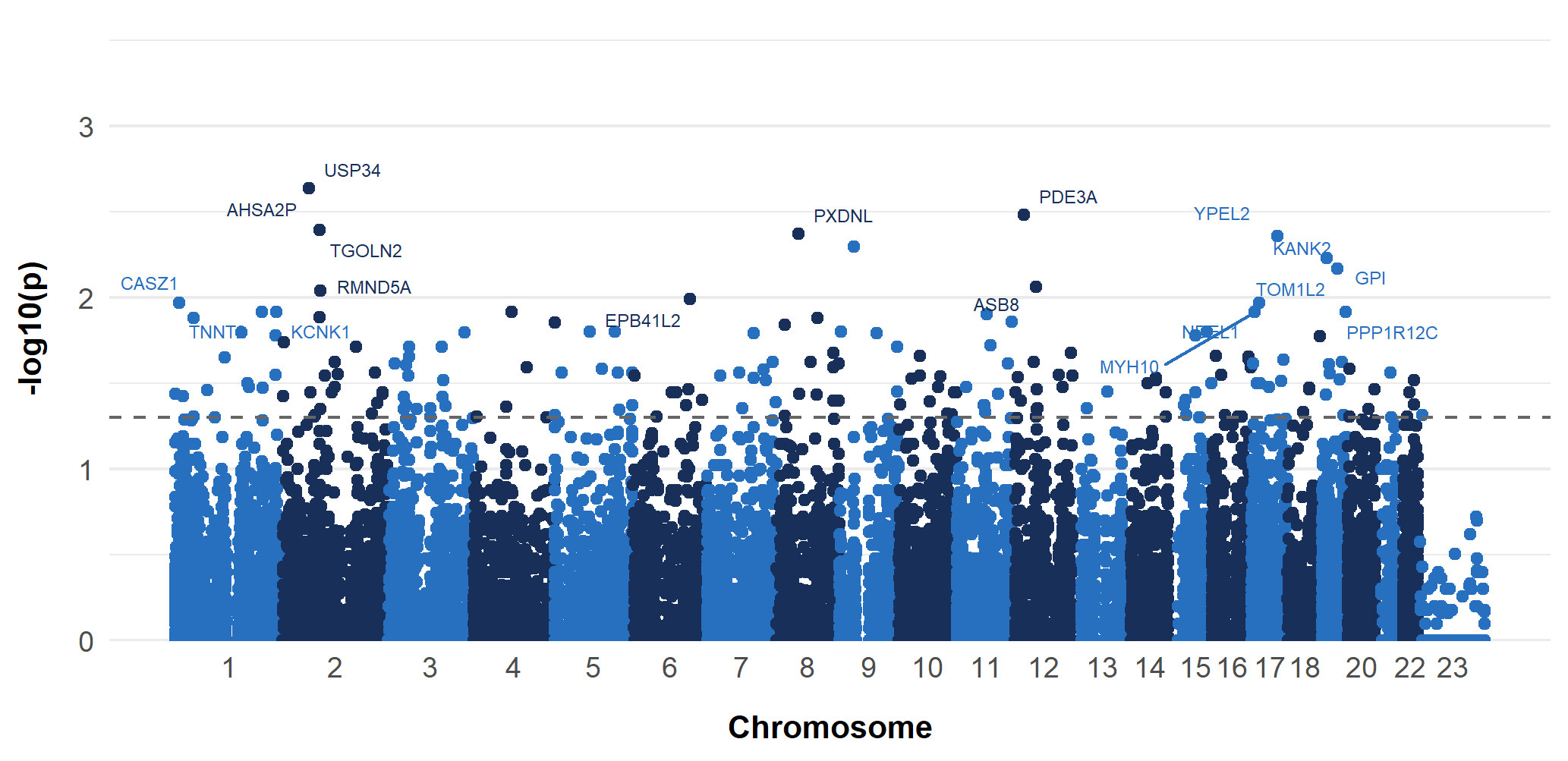

Supplement: Supplementary file 2 — Supplementary Information 2. [file 41598_2023_27591_MOESM2_ESM.zip › Group_4vs2_results/Group_4vs2_Manhattan.tif]

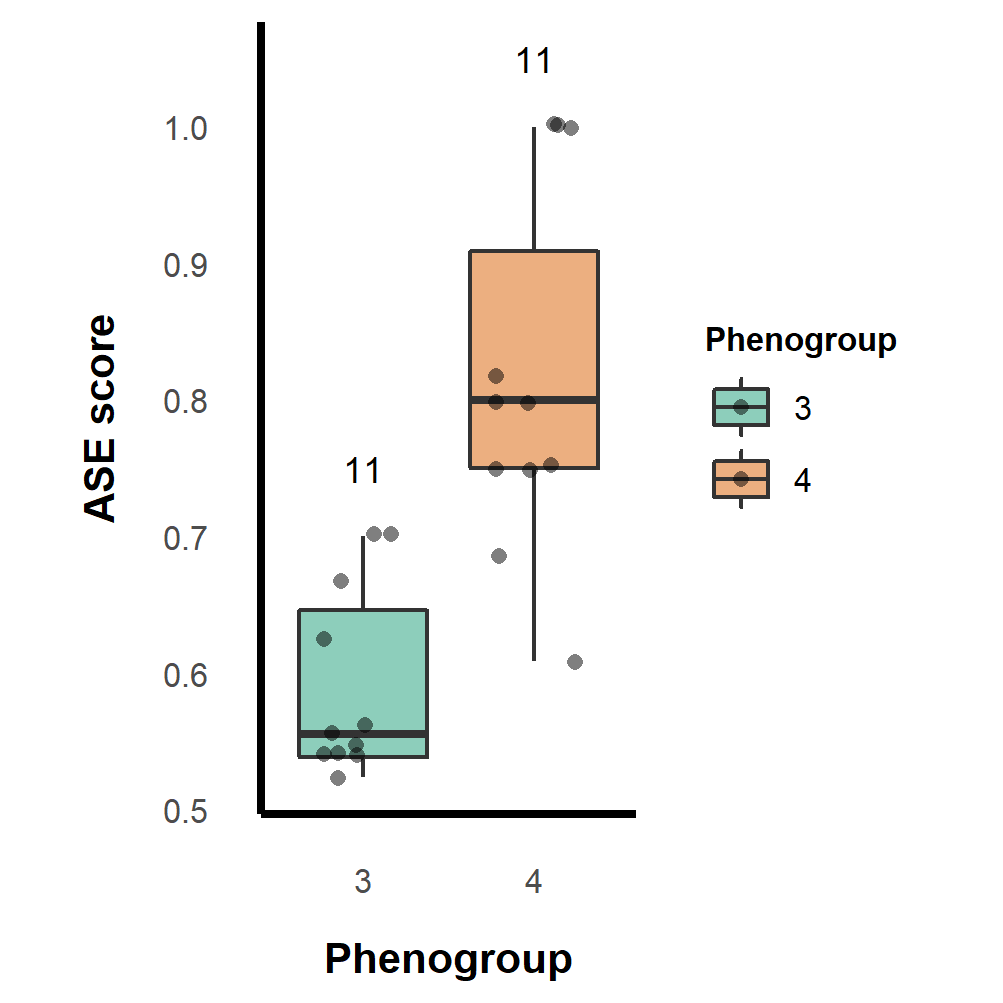

Supplement: Supplementary file 2 — Supplementary Information 2. [file 41598_2023_27591_MOESM2_ESM.zip › Group_4vs3_results/Boxplot_rs9766_across.tif]

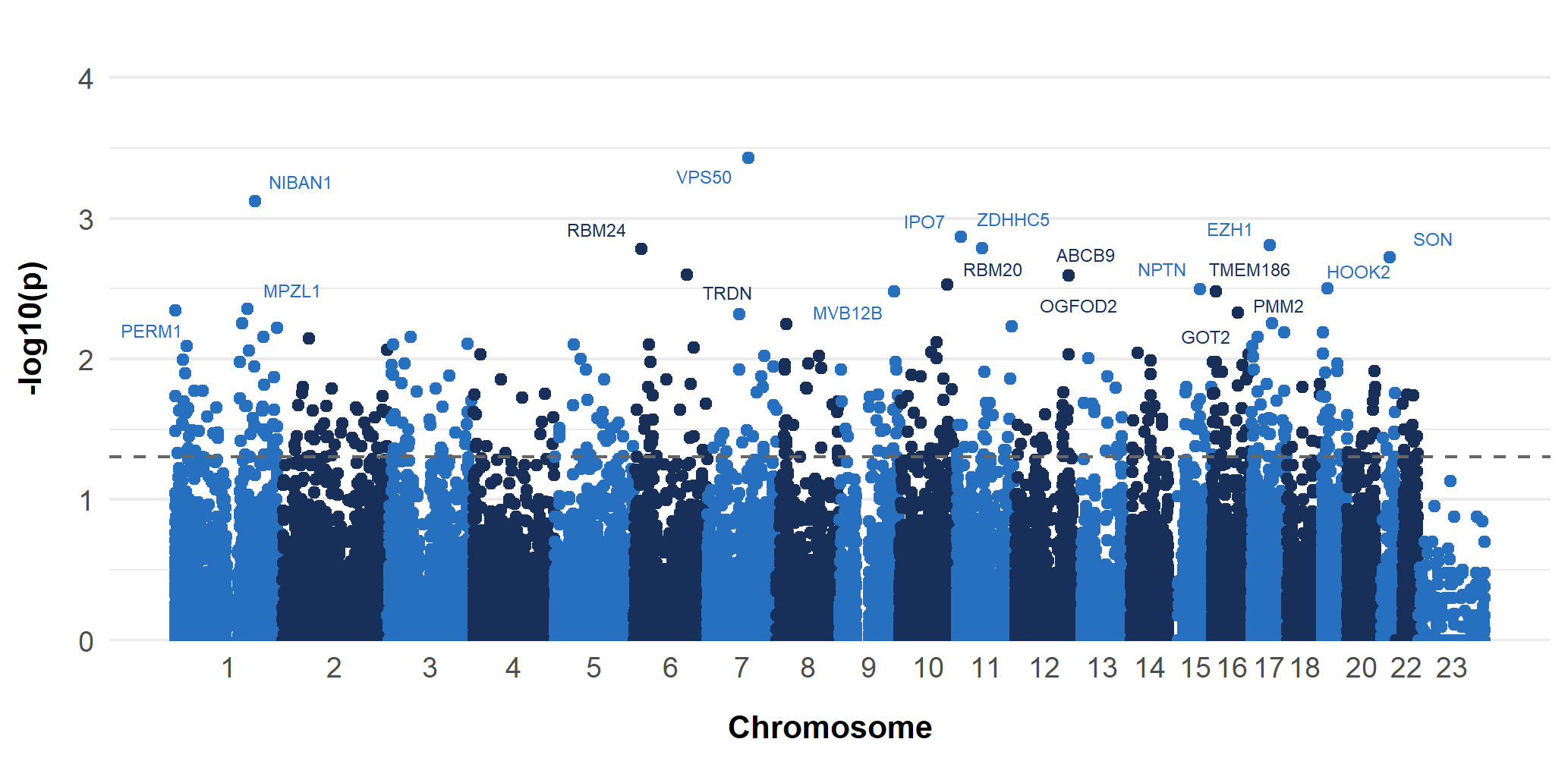

Supplement: Supplementary file 2 — Supplementary Information 2. [file 41598_2023_27591_MOESM2_ESM.zip › Group_4vs3_results/Group_4vs3_Manhattan.tif]

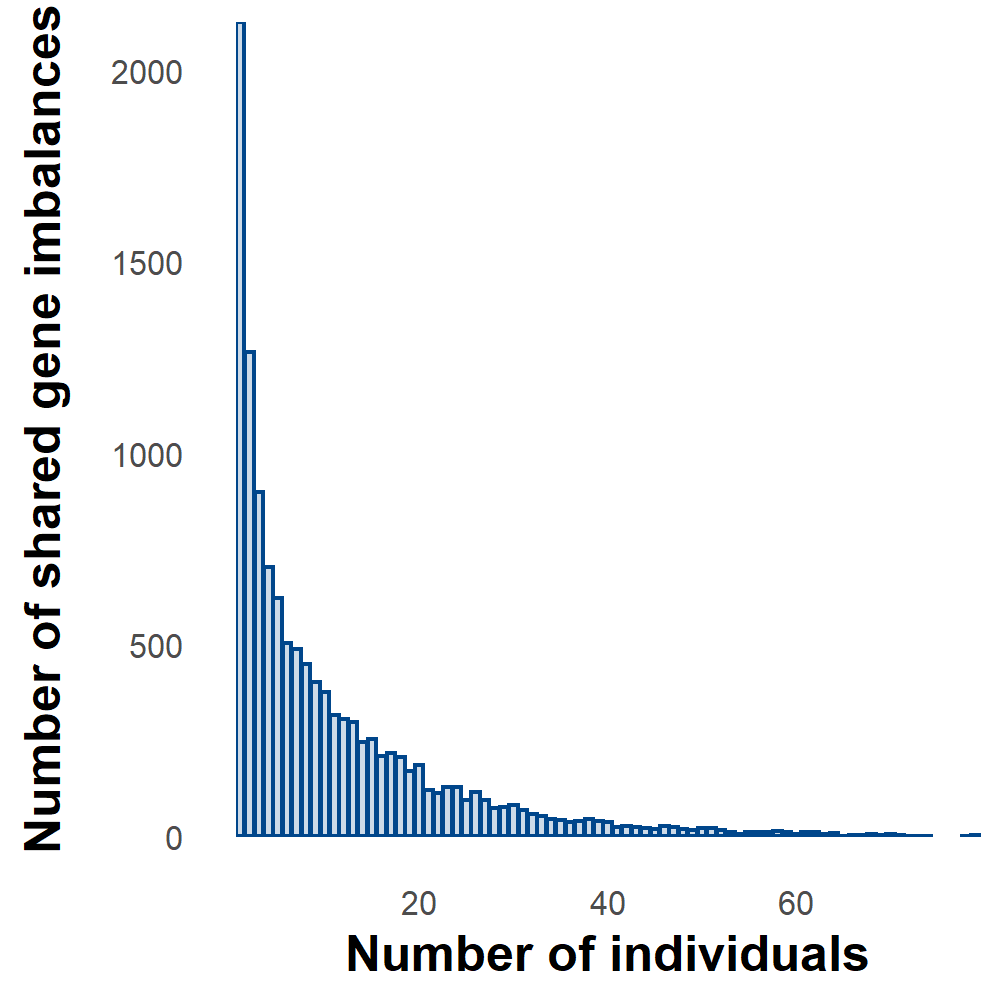

Supplement: Supplementary file 2 — Supplementary Information 2. [file 41598_2023_27591_MOESM2_ESM.zip › Supplementary_Figure1.tif]

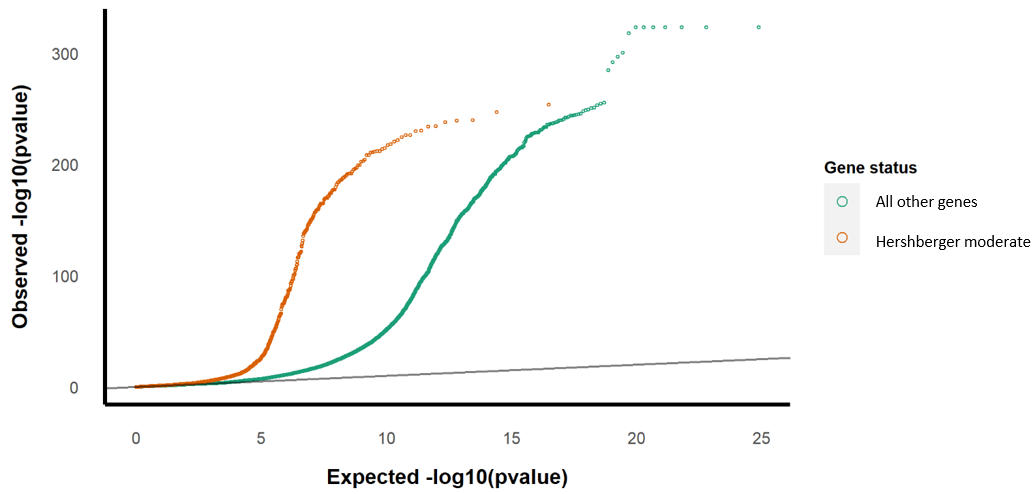

Supplement: Supplementary file 2 — Supplementary Information 2. [file 41598_2023_27591_MOESM2_ESM.zip › Supplementary_Figure2.png]

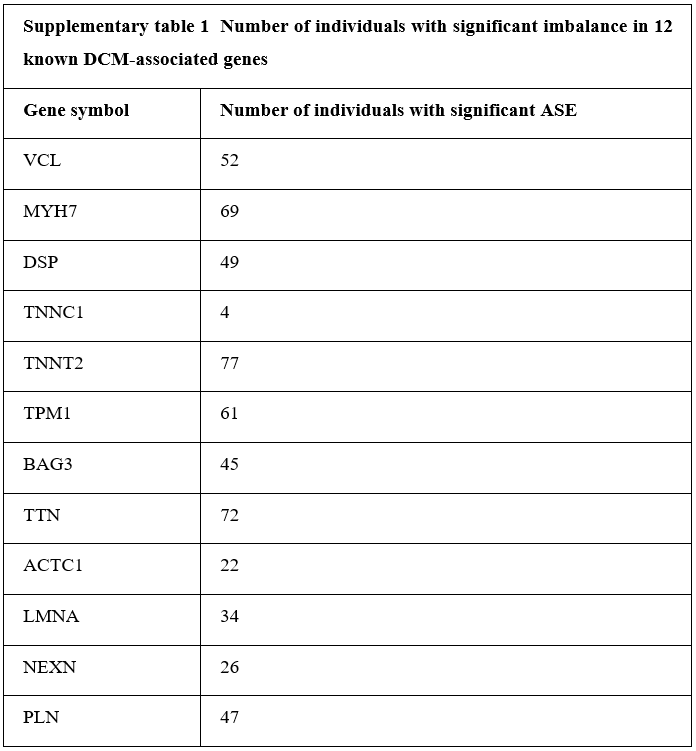

Supplement: Supplementary file 2 — Supplementary Information 2. [file 41598_2023_27591_MOESM2_ESM.zip › Supplementary_Table1.tif]
